# The evolutionary dynamics of endemic human coronaviruses

**DOI:** 10.1093/ve/veab020

**Published:** 2021-03-20

**Authors:** Wendy K Jo, Christian Drosten, Jan Felix Drexler

**Affiliations:** 1 Charité–Universitätsmedizin Berlin, corporate member of Freie Universität Berlin and Humboldt-Universität zu Berlin, Institute of Virology, Berlin, Germany; 2 German Centre for Infection Research (DZIF), associated partner Charité-Universitätsmedizin Berlin, Berlin, Germany

**Keywords:** mutations, genetic variability, evolutionary rate, human coronaviruses, vaccine

## Abstract

Community protective immunity can affect RNA virus evolution by selecting for new antigenic variants on the scale of years, exemplified by the need of annual evaluation of influenza vaccines. The extent to which this process termed antigenic drift affects coronaviruses remains unknown. Alike the severe acute respiratory syndrome coronavirus-2 (SARS-CoV-2), seasonal human coronaviruses (HCoV) likely emerged from animal reservoirs as new human pathogens in the past. We therefore analyzed the long-term evolutionary dynamics of the ubiquitous HCoV-229E and HCoV-OC43 in comparison with human influenza A virus (IAV) subtype H3N2. We focus on viral glycoprotein genes that mediate viral entry into cells and are major targets of host neutralizing antibody responses. Maximum likelihood and Bayesian phylogenies of publicly available gene datasets representing about three decades of HCoV and IAV evolution showed that all viruses had similar ladder-like tree shapes compatible with antigenic drift, supported by different tree shape statistics. Evolutionary rates inferred in a Bayesian framework were 6.5 × 10^−4^ (95% highest posterior density (HPD), 5.4–7.5 × 10^−4^) substitutions per site per year (s/s/y) for HCoV-229E spike (S) genes and 5.7 × 10^−4^ (95% HPD, 5–6.5 × 10^−4^) s/s/y for HCoV-OC43 S genes, which were about fourfold lower than the 2.5 × 10^−3^ (95% HPD, 2.3–2.7 × 10^−3^) s/s/y rate for IAV hemagglutinin (HA) genes. Coronavirus S genes accumulated about threefold less (*P* < 0.001) non-synonymous mutations (dN) over time than IAV HA genes. In both IAV and HCoV, the average rate of dN within the receptor binding domains (RBD) was about fivefold higher (*P* < 0.0001) than in other glycoprotein gene regions. Similarly, most sites showing evidence for positive selection occurred within the RBD (HCoV-229E, 6/14 sites, *P* < 0.05; HCoV-OC43, 23/38 sites, *P* < 0.01; IAV, 13/15 sites, *P* = 0.08). In sum, the evolutionary dynamics of HCoV and IAV showed several similarities, yet amino acid changes potentially representing antigenic drift occurred on a lower scale in endemic HCoV compared to IAV. It seems likely that pandemic SARS-CoV-2 evolution will bear similarities with IAV evolution including accumulation of adaptive changes in the RBD, requiring vaccines to be updated regularly, whereas higher SARS-CoV-2 evolutionary stability resembling endemic HCoV can be expected in the post-pandemic stage.

## 1. Introduction

Since the beginning of the coronavirus disease 2019 (COVID-19) pandemic, millions of human cases have been reported globally (WHO 2020). COVID-19 is caused by the newly emerged severe acute respiratory syndrome coronavirus-2 (SARS-CoV-2) (Li et al. 2020). By December 2020, >150 vaccine candidates are under development, and several vaccines have concluded phase III trials and licensed in several countries (Corum et al. 2020). Most of the COVID-19 vaccine candidates are mRNA-, subunit-, or vector-based vaccines encoding the spike (S) protein (Folegatti et al. 2020; Jackson et al. 2020; Mulligan et al. 2020; Zhu et al. 2020), which is the surface protein employed by coronaviruses for binding to and entry into the host cell. Thus, SARS-CoV-2 evolution engendering changes of the S protein can have an impact on long-term usability of COVID-19 vaccines.

Within the viral family *Coronaviridae*, viruses infecting humans belong to the genera *Alpha*- and *Betacoronavirus*. SARS-CoV-2 belongs to the *SARS-related coronavirus* species within the genus *Betacoronavirus* (Gorbalenya et al. 2020). All coronaviruses share an unusually long single-stranded RNA genome encompassing 27–32 kb and a similar genomic structure (Su et al. 2016). Error-prone RNA-dependent RNA polymerases (RdRp) of RNA viruses such as SARS-CoV-2 contribute to short generation times and high mutation rates (Domingo 1997; Duffy et al. 2008). However, differently from other RNA viruses, RdRp-driven mutation in coronaviruses is limited by a virus-encoded proofreading protein termed nsp14 (Denison et al. 2011).

Beyond the recently emerged SARS-CoV-2 and MERS-CoV, which emerged in 2012/2013 and causes zoonotic infections predominantly on the Arabian Peninsula, there are four endemic human coronaviruses (HCoV). HCoV-229E and HCoV-OC43 were identified already in the mid-1960s, whereas HCoV-NL63 and HCoV-HKU1 were identified more recently in 2004 and 2005 due to increased screening for HCoV in the aftermath of the SARS epidemic during 2003–2004 (Corman et al. 2018). The endemic HCoV and SARS-CoV-2 share several epidemiological and ecological traits. First, the endemic HCoV are comparable to SARS-CoV-2 in their high transmissibility and worldwide spread (Owusu et al. 2014; Corman et al. 2018; Goes et al. 2020). HCoV cause about 10 per cent of all common colds globally, predominantly during fall and winter seasons, and afford seroprevalence rates of up to 90 per cent already in young children (Corman et al. 2018; Edrige et al. 2020). Second, alike SARS-CoV-2 and many other respiratory viruses, repeated upper respiratory tract infections with HCoV are possible despite prior exposure and detectable systemic immune responses (Callow et al. 1990; Farag et al. 2015), as was exemplified by HCoV-229E re-infection within one year in an experimental infection study in humans (Callow et al. 1990). Third, alike SARS-CoV-2 that has likely evolutionary origins in bats, the endemic HCoV originated from an animal source, including bats, rodents and intermediate hosts (Vijgen et al. 2006; Pfefferle et al. 2009; Huynh et al. 2012; Corman et al. 2015; Jo et al., 2020).

Vaccines against COVID-19 provide a powerful mean to create herd immunity and control the pandemic, albeit long-term efficacy remains to be determined (Krammer 2020). No vaccine has yet been approved against any other HCoV. On the contrary, there are several vaccines against the major respiratory illness influenza, which is likely comparable to COVID-19 in the potential for pandemic spread and disease severity (Petersen et al. 2020). Influenza A viruses (IAV) have high evolutionary rates (Fitch et al. 1991) and evolve into antigenically distinct variants escaping community protective immunity within a few years, a process that is termed antigenic drift and requires evaluation and sometimes exchange of vaccine strains on an annual basis (Carrat and Flahault 2007; Rambaut et al. 2008). Among IAV, the endemic subtype H3N2 exhibits the strongest antigenic drift (Fitch et al. 1991; Bedford et al. 2015), largely influenced by its higher mutation rate (Pauly et al. 2017) and large effective population size (Rambaut et al. 2008). Once sufficient community immune responses against SARS-CoV-2 have been built either by wild-type infection or vaccination, a plausible post-pandemic scenario would be that the future trajectory of SARS-CoV-2 will be reminiscent of endemic HCoV and IAV (Petersen et al. 2020). Under this premise, we investigated the genetic variation of two prototypic endemic HCoV in comparison to IAV H3N2 by analyzing tree shape statistics, evolutionary rates and selection pressure using publicly available datasets encompassing more than thirty years of coronavirus and influenza virus evolution.

## 2. Materials and methods

### 2.1 Sequence data

Complete genes encoding the S glycoproteins of HCoV-229E, HCoV-NL63, HCoV-HKU1 and HCoV-OC43 were downloaded from GenBank via Geneious v11.1.5 (https://www.geneious.com). Complete hemagglutinin (HA) gene sequences of IAV subtype H3N2 circulating from 1991 to 2019 from the FLU project were downloaded from the NCBI Influenza virus database (https://www.ncbi.nlm.nih.gov/genomes/FLU). HA sequences were chosen from northern temperate regions as most of the recent HCoV sequences available in GenBank were from those regions. Duplicated gene sequences were removed using the function ‘find duplicates’ in Geneious. Translation alignments of each dataset were performed using the MAFFT (Katoh and Standley 2013) plugin with an iterative refinement algorithm G-INS-i implemented within Geneious.

### 2.2 Maximum likelihood phylogenies

Maximum likelihood (ML) phylogenies of the complete S coding sequence datasets of HCoV-229E, HCoV-NL63, HCoV-HKU1, and HCoV-OC43, as well as the HA coding sequence dataset of IAV H3N2 were reconstructed using IQ-TREE (Nguyen et al. 2015) with 1,000 ultrafast bootstrap replicates (UFBoot) (Hoang et al., 2018) and 1,000 Shimodaira–Hasegawa approximate likelihood test (SH-aLRT) (Anisimova et al. 2011) for statistical support of grouping. Gaps were treated as missing data and removed from the analyses. The best-fit nucleotide substitution model was TIM + F + I + G4 (transition model with variable base frequencies, variable transition rates and two transversion rates), according to the Bayesian information criterion yielded by ModelFinder for the HCoV-OC43 S dataset (Kalyaanamoorthy et al. 2017), which corresponded to the largest HCoV dataset available in GenBank and was used subsequently for all datasets to enhance comparability. Notably, TIM + F + I + G4 was also one of the most supported substitution models for HCoV-229E (ranked third by ModelFinder), suggesting robustness of its usage for HCoV datasets. All tree files were visualized with FigTree from the BEAST package (Suchard et al. 2018).

### 2.3 Recombination analyses

Recombination analyses were carried out with the methods RDP, GENECONV, Bootscan, MaxChi, Chimaera, SiScan and 3Seq implemented in RDP4 (Martin et al. 2015). Sequences with predicted recombination events that were detected with more than two methods and *P* < 0.05 were excluded. Sequences with recombination events supported by less than three methods were analyzed individually and excluded from downstream analyses only if ML phylogenies of genomic regions adjacent to predicted breakpoints showed statistically supported different topologies.

### 2.4 Temporal signal

The temporal signal (clock-likeliness) of the data was evaluated using a linear regression of root-to-tip genetic distances against sampling time in TempEst v1.5.3 (Rambaut et al. 2016). Correlation coefficients and *R*^2^ were calculated using the function heuristic residual mean squared and best-fitting root option. In addition, the clock-likeliness of HCoV-229E and HCoV-OC43 datasets were evaluated with Bayesian dating permutations. To this end, ten datasets with randomly permuted sampling dates were created using the R library TipDatingBeast (Rieux and Khatchikian 2017) and the estimated evolutionary rates of the original dataset were compared with date-randomized datasets. Sufficient temporal signal in a dataset was defined following the criterion that the 95 per cent highest posterior density (HPD) intervals of the evolutionary rate estimate of the original dataset do not overlap with those generated using date-randomized datasets (Duchene et al. 2015). Bayesian dating permutations were done using Beast v2.6.3 (Bouckaert et al. 2019). Analyses were run for 50 million generations, with sampling every 5000 steps.

### 2.5 Clock rate

The evolutionary rates in substitutions per site per year (s/s/y) with 95 per cent HPD intervals of the glycoprotein gene datasets calibrated by sampling years were estimated using Beast2. The Nested Sampling algorithm (Maturana Russel et al. 2019) was used to compare the marginal likelihoods of three clock models: strict-clock, exponential relaxed-clock and lognormal relaxed-clock, as well as three coalescent tree priors: constant population growth, exponential population growth and Bayesian skyline for the HCoV-OC43 S gene dataset. A model was considered to be strongly favored if logarithmized Bayes factors (BF) were more than two (Kass and Raftery 1995). Bayesian model averaging was used to infer the most appropriate substitution model for the HCoV-OC43 S gene dataset via the bModelTest package (Bouckaert and Drummond 2017), implemented in Beast2. The final analyses were run for 50 million generations with ten per cent burn-in, sampling every 5,000 steps, applying the most appropriate settings: TIM + G4 + I as substitution model, a strict clock (uniform prior between 0 and 1, BF >350) and an exponential growth coalescent tree prior (BF >550) using default prior distributions. The same parameters were used for all three datasets to enhance comparability. The HCoV-OC43-best fit substitution model was also the second-best substitution model for HCoV-229E according to bModelTest. Additional analyses were performed using a lognormal relaxed-clock model with parameters as described above.

### 2.6 Tree shape statistics

Phylogenetic tree shapes of ML and Bayesian trees were compared using different tree metrics for imbalance or asymmetry, including the Colless index, the Sackin index, the number of cherries, average ladder length, number of internal nodes, and the staircase-ness. All values were calculated and normalized using the R package phyloTop (Kendall et al. 2018). Normalization consisted in the division of maximum possible number of tips (Kendall et al. 2018). The Colless and Sackin indices measure overall asymmetry in a tree, number of cherries count the number of branches with two tip descendants, average ladder length is defined by the mean size of ladders in a tree, being ladder a series of connected internal nodes with one leaf descendant, number of internal nodes with a single tip (Colijn and Gardy 2014), and staircase-ness measures the proportion of subtrees that are imbalanced in the proportion of taxa descending from ancestral nodes (Norstrom et al. 2012).

### 2.7 Mutation detection

Non-synonymous substitutions (dN) and insertion-deletion mutation (indel) rates for each codon site were calculated using the Nei-Gojobori method in SNAP v2.1.1 (www.hiv.lanl.gov). To determine the cumulative dN from 2001 to 2019, average rates of dN in the glycoprotein genes were calculated and compared between viruses using a two-way analysis of variance with Tukey post hoc tests using GraphPad Prism v6 (La Jolla, CA, USA, www.graphpad.com). Unpaired *t*-tests with Welch correction were used to compare average rates of dN within and outside of the receptor binding domain (RBD) in GraphPad Prism v6.

### 2.8 Selection pressure analyses

Selection pressure analyses were performed using the software packages Phylogenetic Analysis by Maximum Likelihood (PAML) (Yang 2007) and HyPhy in Datamonkey.org (Pond et al. 2005) in datasets encompassing HCoV S gene sequences after exclusion of sequences with evidence for recombination. For HCoV-OC43, S sequences considered as outliers identified by root-to-tip regression analyses were excluded for pressure analyses (Supplementary Table S1). For IAV H3N2, pressure analyses were conducted in a subset of HA sequences from the FLU project belonging to the years 1991–2019, available at NCBI influenza virus database, with mutual sequence identity of <99.5 per cent generated using CD-HIT-EST (Supplementary Table S1) (Huang et al. 2010). In PAML, statistical tests were performed using the CodeML program (Xu and Yang 2013). The codon-substitution models M7 (beta) and M8 (beta and ω) were used to analyze the datasets using an F61 codon frequency model. Triplet codon gaps and ambiguities were removed from analyses using the cleandata option. For each dataset, evidence for positive selection was evaluated by calculating likelihood-ratio tests of the site-specific models M7 vs. M8. Statistical significance was assessed using a chi-square (χ^2^) distribution with two degrees of freedom. Sites were considered under positive selection if significance levels were *P* < 0.05 and posterior probability above >0.9 in BEB under the model M8. In HyPhy, the ML-based methods MEME, SLAC, and FUBAR were used to detect sites under positive selection. The best substitution model was selected automatically, and sites were considered under selection if *P* < 0.1 for ML methods or if the posterior probability was >0.9 for FUBAR. To compare the number of sites under positive selection between different regions along the analyzed genes, χ^2^ tests were performed using GraphPad Prism v6.

## 3. Results

### 3.1 Recombinant and low-quality sequences distort the temporal signal of HCoV

**Figure 1.**
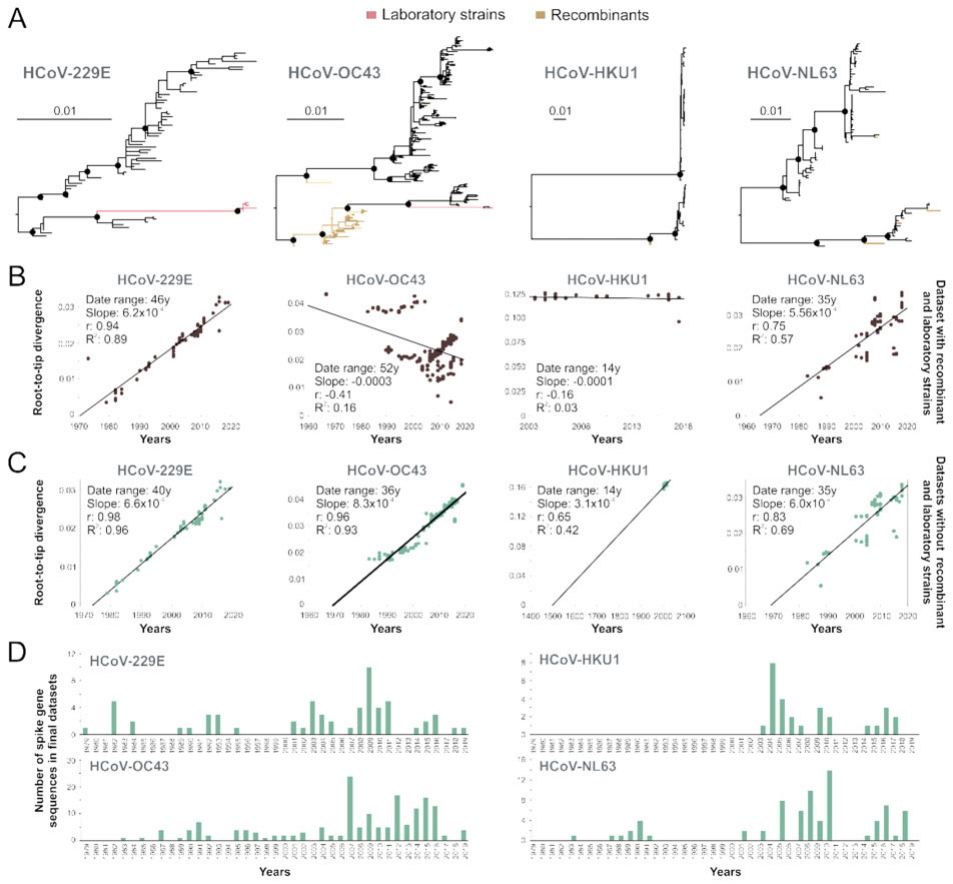
Phylogeny of endemic human coronaviruses. (A) ML phylogenies of complete glycoprotein genes of HCoV-229E, HCoV-OC43, HCoV-HKU1, and HCoV-NL63. Circles at nodes indicate support of ≥80 SH-alrt/≥95 UFBoot for major clades. Scale bars indicate number of nucleotide substitutions per site. (B) Linear regression of root-to-tip genetic distances over time in years of whole datasets. (C) Linear regression plots of HCoV datasets excluding recombinant sequences and laboratory strains. The date range, slope (rate), correlation coefficient, and R^2^ are shown in the graph. (D) Number of HCoV spike gene sequences per year after exclusion of recombinant and laboratory strains retrieved from NCBI (detailed in Supplementary Table S1).

**Figure 2.**
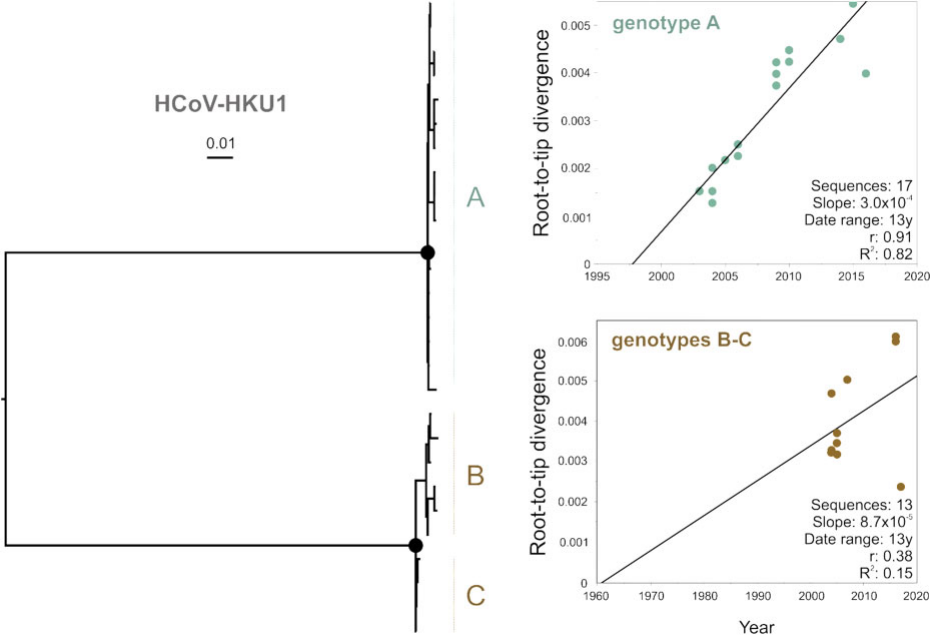
Linear regression plots of root-to-tip divergence over time of major sub-lineages of HCoV-HKU1. Circles at nodes in the ML phylogeny relying on complete spike genes indicate support of ≥80 SH-alrt/≥95 UFBoot for major clades. Scale bars indicate number of nucleotide substitutions per site. Linear regression of root-to-tip genetic distances over time in years after exclusion of recombinant sequences (detailed in Table S1). The date range, slope (rate), correlation coefficient (r), and R^2^ are shown in the graph.

Publicly available sequence information may not contain adequate information on time of isolation and recombinant sequences can affect evolutionary reconstructions (Rasche et al. 2019). Using all publicly available sequences, temporal analyses of HCoV phylogenies (Fig. 1A) indicated poor temporal signal with *R*^2^ values of 0.03 for HCoV-HKU1, 0.16 for HCoV-OC43, 0.57 for HCoV-NL63 and 0.89 for HCoV-229E (Fig. 1B). Poor temporal signal was consistent with the existence of recombinant sequences or other sequences confounding the temporal analyses (Fig. 1A, Supplementary Table S1). For HCoV-OC43, most recombination events detected were located in sequences belonging to a clade, which has been previously reported as a recombinant genotype tentatively termed genotype E (Zhang et al. 2015). After excluding likely recombinant sequences and HCoV prototype strains likely containing mutations that may have arisen during multiple passages in different cell cultures and even animals and whose sampling dates are uncertain, the temporal signal of those datasets increased as suggested by the change of *R*^2^ from 0.89 to 0.96 for HCoV-229E, from 0.16 to 0.93 for HCoV-OC43, from 0.03 to 0.42 for HCoV-HKU1, and from 0.57 to 0.69 for HCoV-NL63 (Fig. 1C). Subsequently, we decided to exclude HCoV-HKU1 and HCoV-NL63 from downstream evolutionary analyses. In the case of HCoV-HKU1, the dataset had insufficient temporal signal likely due to the limited number of sequences (Fig. 1D) and limited genetic divergence over time. Beyond the small number of available sequences, there were differences in their clock-like evolution between the two major clades of HCoV-HKU1, particularly the clade encompassing genotypes B and C (Fig. 2). The reason for the apparently different evolutionary structure of HCoV-HKU1 genotypes remains to be determined. HCoV-NL63 was excluded from downstream analyses because sequence coverage over time was inadequate, lacking sequences from the entire 1990s (Fig. 1D, Supplementary Table S1). The final HCoV datasets used for downstream analyses consisted of 62 S gene sequences of HCoV-229E and 169 S gene sequences of HCoV-OC43 (Fig. 1D). The time span of collected sequences in final datasets ranged from 1979 to 2019 for HCoV-229E S genes, and from 1983 to 2019 for HCoV-OC43 S genes. For comparison, we selected one sequence per location per year of complete HA encoding sequences of IAV H3N2 sampled from 1991 to 2019, leading to a dataset encompassing 477 HA sequences (Supplementary Table S1). A root-to-tip regression analysis of the IAV H3N2 HA dataset used in this study yiedled a strong temporal signal (*R*^2^ = 0.99) (Supplementary Fig. S1), as previously reported for H3N2, suggesting robustness of our data (Westgeest et al. 2012).

### 3.2 The phylogenies of HCoV-229E and HCoV-OC43 are compatible with antigenic drift

**Figure 3.**
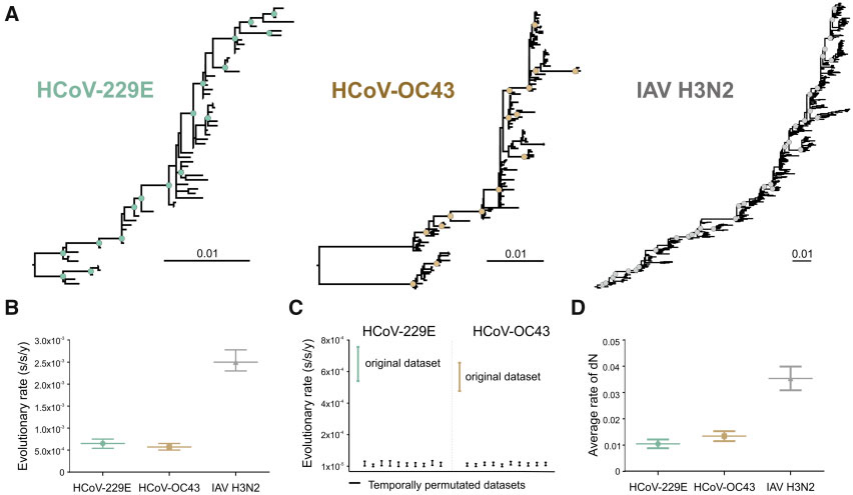
Evolution of HCoV-229E , HCoV-OC43 and IAV H3N2 over time. (A) Maximum likelihood phylogenies relying on complete viral glycoprotein datasets. Circles at nodes indicate support of ≥80 SH-alrt/≥95 UFBoot for major clades. Scale bars indicate number of nucleotide substitutions per site. Sequences used are detailed in Supplementary Table S1. (B) Evolutionary rates in substitution per site per year (s/s/y) with 95 per cent HPD intervals inferred in a Bayesian framework relying on complete viral glycoprotein datasets. (C) Comparison between the 95 per cent HPD intervals of the evolutionary rates of the HCoV-229E S (teal) final dataset with date-randomized datasets (*n* = 10, black), and of the HCoV-OC43 S (mustard) final dataset with date-randomized datasets (*n* = 10, black). (D) Average rate of non-synonymous substitutions (dN ± SEM) for HCoV-229E S, HCoV-OC43 S, and IAV H3N2 HA from 2001 to 2019.

The shape of a phylogenetic tree can inform on the evolutionary forces acting on the taxa that are analyzed. The tree shape of both HCoV-229E and HCoV-OC43 S-based reconstructions showed a ladder-like shape with long trunks and short terminal branches, which was compatible with antigenic drift and comparable to the IAV H3N2 HA-based reconstructions (Fig. 3A). The IAV tree shape was consistent with previous analyses of H3N2 evolution (Fitch et al. 1991), suggesting robustness of our data. Ladder-like phylogenies are characterized by the replacement of one variant by another usually due to antigenic drift (Frost and Volz 2013), producing an imbalanced or asymmetric tree (Gray et al. 2011). The ladder-like shape in all three datasets was supported by various tree shape statistics (Table 1). Similar values were obtained for cherry configuration, number of internal nodes, ladder length and staircase-ness for both ML and Bayesian trees. A staircase-ness of about 0.7 indicated a high proportion of imbalanced subtrees in all three datasets. In contrast, the Colless and Sackin indices indicated that the HCoV-229E tree shape was overall more imbalanced than in the case of HCoV-OC43 and IAV H3N2, possibly due to the fewer number of HCoV-229E sequences distributed over time (Fig. 1D). To control for potential biases from uneven numbers of sequenecs available for different viruses over time, analyses were also performed on a reduced dataset (Supplementary Table S1) consisting of only one sequence per year per location. Both tree shape and tree metrics (Supplementary Fig. S1, Table S2) were compatible with antigenic drift, suggesting robustness of the results.

**Table 1.** Tree shape statistics.

Tree statistic	Description	HCoV-229E		HCoV-OC43		IAV H3N2	
		ML	B	ML	B	ML	B
Colless index^a^	Assess overall asymmetry	0.42	0.44	0.18	0.16	0.24	0.22
Sackin index^a^	Assess overall asymmetry	0.50	0.52	0.23	0.21	0.26	0.23
Cherry number	Count branches with two tips	0.54	0.52	0.51	0.53	0.57	0.59
Number internal nodes	Count internal nodes with a single tip child	0.49	0.50	0.47	0.47	0.43	0.42
Ladder length	Measures mean size of ladders	2.44	3.11	2.5	2.53	2.5	2.38
Staircase-ness^a^	Count proportion of imbalanced subtrees	0.72	0.72	0.72	0.69	0.69	0.67

a Value of 1 indicates perfect asymmetry, value of 0 indicates perfect symmetry (Colijn and Gardy 2014).

B, Bayesian.

### 3.3 HCoV have lower evolutionary rates than IAV H3N2

On a short time scale, RNA viruses typically show very high evolutionary rates, but those rates can differ more than tenfold between RNA virus families (Jenkins et al. 2002). In this study, the evolutionary rates of the HCoV S genes were estimated to be 6.5 × 10^−4^ (95% HPD, 5.4–7.5 × 10^−4^) s/s/y for HCoV-229E and 5.7 × 10^−4^ (95% HPD, 5–6.5 × 10^−4^) s/s/y for HCoV-OC43 using a strict clock model and an exponential growth coalescent tree prior (Fig. 3B). Evolutionary rates did not vary greatly when using a different clock model ( uncorrelated lognormal relaxed clock; Supplementary Table S3), suggesting robustness of our results. The evolutionary rate for HCoV-229E inferred here was higher than previously reported for HCoV-229E at 4.3 × 10^−4^ s/s/y (Al-Khannaq et al. 2016), whereas the evolutionary rate estimated for HCoV-OC43 was on the lower limit of the range of previously reported values at 5.8–8.5 × 10^−4^ s/s/y (Lau et al. 2011; Ren et al. 2015; Oong et al. 2017). The minor differences between the rate estimates that were previously reported and the estimates generated in this study are likely due to different datasets from different time spans and locations as well as substitution models used in those analyses. For IAV H3N2, we obtained an evolutionary rate of 2.5 × 10^−3^ (95% HPD, 2.3–2.7 × 10^−3^) s/s/y (Fig. 3B), which was comparable to the 1.5 × 10^−3^ s/s/y estimated previously using a HA gene dataset from the Middle East and North Africa (Al Khatib et al. 2019), and only slightly lower than the 4.2–5.2 × 10^−3^ s/s/y estimated when only analyzing the more variable HA1 gene subunit (Nyang'au et al. 2020; Westgeest et al. 2012), again suggesting robustness of our results. In sum, IAV H3N2 had an about fourfold higher evolutionary rate compared with HCoV-OC43 and HCoV-229E. The lower HCoV evolutionary rate compared with IAV and other RNA viruses can likely be attributed to the lower mutation rate in coronaviruses due to nsp14-mediated proofreading activity (Denison et al. 2011; Peck and Lauring 2018). This characteristic proofreading activity in coronaviruses and other members of the order *Nidovirales* has been hypothesized to contribute to their larger genome sizes of more than 26 kilobases (kb) compared with other RNA viruses, such as influenza viruses whose genomes encompass only about 13.5 kb (Peck and Lauring 2018). Genome sizes have been negatively correlated to evolutionary rates (Sanjuan 2012), suggesting robustness of our results. Notably, relatively lower evolutionary rates of HCoV are not necessarily at odds with the 1 × 10^−3^ s/s/y that are currently estimated for SARS-CoV-2 on the complete genome-level (Boni et al. 2020), because the high SARS-CoV-2 rate is very likely to decrease over time due to purifying selection (Duchene et al. 2014).

None of the 95 per cent HPD intervals of the estimated evolutionary rates of date-randomized datasets for HCoV-229E (2.6 × 10^−7^–3.3 × 10^−5^ s/s/y) and HCoV-OC43 (1.1 × 10^−6^–2.5 × 10^−5^ s/s/y) overlapped with the 95 per cent HPD interval of the estimated rate of the original dataset of HCoV-229E and HCoV-OC43 reported above (Fig. 3C), confirming that the datasets had sufficient temporal signal to permit adequate rate estimates and downstream analyses (Duchene et al. 2015).

### 3.4 HCoV accumulate less amino acid changes over time than IAV H3N2

Amino acid changes in viral proteins can lead to immune escape due to a decrease in the ability of the adaptive immune response raised by a primary infection or vaccination to control a second infection with the same virus (Drexler et al. 2014; Linderman et al. 2014; Romano et al. 2015). To quantitate the amount of mutations generating amino acid changes, we calculated the cumulative rate of dN per genome position from 2001 to 2019 for each virus dataset (Fig. 3D). Both HCoV-229E S and HCoV-OC43 S had a significantly overall lower rate of dN compared to IAV H3N2 HA (approximately threefold less; *P* < 0.0001).

### 3.5 Non-synonymous mutations predominantly occur in the RBD of HCoV and IAV

**Figure 4.**
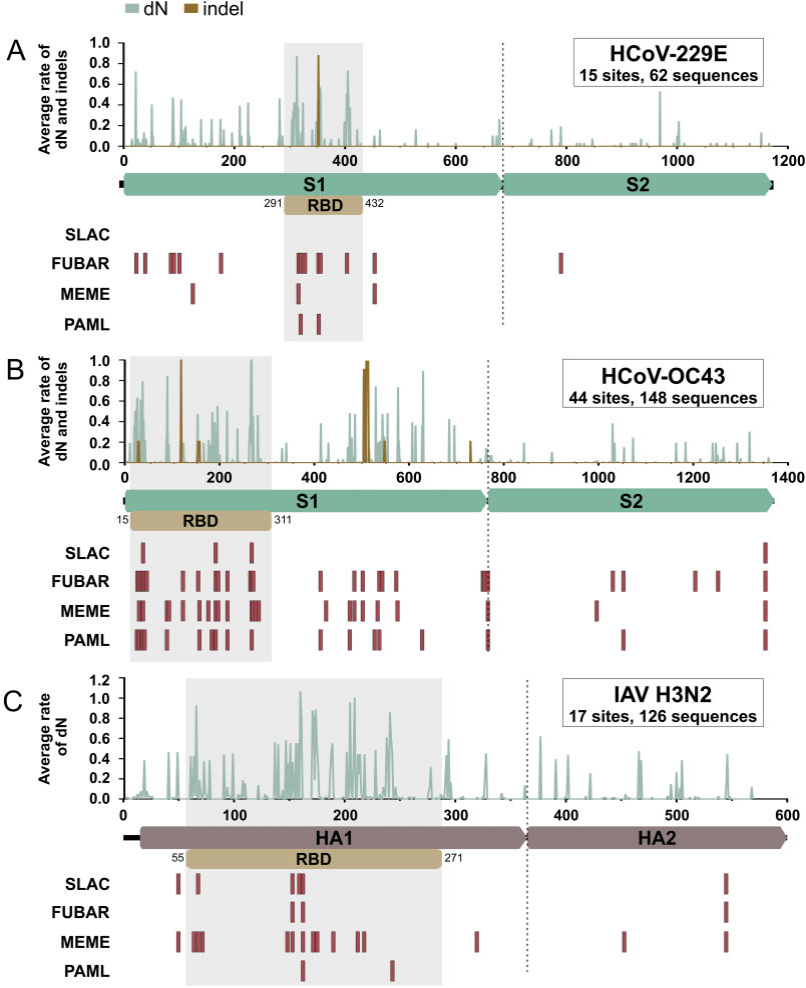
Mutations and sites under positive selection in endemic HCoV and IAV glycoprotein genes. (A–C) Average rate of non-synonymous mutations (dN) and indel mutations at each position along the glycoprotein genes of HCoV-229E, HCoV-OC43, and IAV H3N2 are depicted in green (dN) and brown (indel). Sites under positive selection are depicted as red bars below gene sketches. The HCoV-229E RBD position is based on GenBank accession no. MH048989 according to Li et al. (2019). The HCoV-OC43 RBD position is based on GenBank accession no. AY903460 according to Hulswit et al. (2019). The IAV H3N2 RBD position is based on GenBank accession no. CY173187 according to DuBois et al. (2011).

The coronavirus S protein contains two subunits, S1 and S2 (Fig. 4). Whereas interaction with the cellular receptor occurs via the S1 subunit at the RBD, membrane fusion occurs via the S2 subunit (Graham and Baric 2010). The RBD of HCoV-229E lies within the domain B of S1 between positions 291 and 432 and binds to human aminopeptidase N (Li et al. 2019), whereas the RBD of HCoV-OC43 lies within the domain A of S1 between positions 15 and 310 and binds to 9-*O*-acetylated sialic acid (Hulswit et al. 2019). The influenza virus HA protein is also divided into two subunits, HA1 and HA2. The HA1 subunit again contains the RBD (Fig. 4), which binds to sialic acids, such as α2,6-linked sialic acid in the case of endemic IAV (DuBois et al. 2011).

Non-synonymous substitutions or indel mutations occurred in 10 per cent of codons of the HCoV-229E S gene, in 16 per cent of codons of the HCoV-OC43 S gene, and in 29 per cent of codons of the IAV H3N2 HA gene (Table 2). Of note, comparisons of the number of dN between the coronaviruses under study should be taken with caution because the HCoV-OC43 dataset was relatively larger than the HCoV-229E dataset (Table 2) and non-recent common ancestry may limit non-independence of datum points. Irrespective of the different dataset sizes, the distribution of dN or indel mutations over the protein was comparable between HCoV and IAV. In HCoV, more than 70 per cent (*P* < 0.0001) of the total amino acid sites with dN or indel mutations were mapped to the S1 subunit, whereas for IAV, about 80 per cent of codons with dN (*P* < 0.0001) were mapped to the HA1 subunit (Table 2). Moreover, of the total dN and indel mutations within the S1 subunits, around 35 to 40 per cent (*P* < 0.05) of all mutations were located within the RBD , whereas for IAV, about 70 per cent (*P* < 0.05) of mutations within the HA1 subunit were located within the RBD (Table 2). In addition to the number of amino acid exchanges occurring within the RBD, the average rates of dN within the RBD were higher than in other regions of the glycoprotein genes by approximately fourfold (*P* < 0.0001) in both HCoV-OC43 and IAV H3N2, and sevenfold (*P* < 0.0001) in HCoV-229E. In sum, these data suggest that the RBD are hot spots for amino acid substitutions potentially representing adaptive evolution in HCoV and IAV.

**Table 2.** Non-synonymous mutations and codons under positive selection.

Virus	No. of sequences	Gene region	Length^a^	dN and indel		Positive selection	
				No. of codons	*P* value*^b^	No. of codons	*P* value*^b^
		S	1172	121		15	
HCoV-229E^b^	59	S1	688	92	<0.0001	14	0.0060
		RBD	142	34	<0.0001	6	0.0380
		S	1362	217		44	
HCoV-OC43^b^	148	S1	766	152	<0.0001	38	<0.0001
		RBD	296	71	0.0225	23	0.0045
		HA	566	165		17	
IAV H3N2^b^	477	HA1	330	134	<0.0001	15	0.0192
		RBD	217	95	0.0143	13	0.0807

a Amino acid residues.

b Gene lengths according to GenBank nos. MH048989 for HCoV-229E, AY903460 for HCoV-OC43, and CY173187 for IAV H3N2.

* Statistical significance according to χ^2^ tests.

indel, insert-deletion mutations; S, spike.

### 3.6 Adaptive evolution predominantly affects the RBD of HCoV and IAV

In IAV, amino acid exchanges conferring escape from adaptive immune responses are predominantly located adjacent to the receptor binding site (Koel et al. 2013), which are those sites within the RBD interacting with the cellular receptor. Community protective immune responses may have left signs of positive selection at sites potentially responsible for antigenic drift in HCoV. Evidence of gene-wide positive selection was found for both the S genes of HCoV-229E and HCoV-OC43 and the HA gene of IAV H3N2 (Table 3). Significantly more sites under positive selection were located within the S1 subunits of HCoV-229E (*P* < 0.001) and HCoV-OC43 (*P* < 0.0001), and within the HA1 in IAV H3N2 (*P* < 0.05) (Fig. 4, Table 2, Supplementary Tables S4–S6). Of those sites under positive selection within the S1 and HA1 subunits, 42.9 per cent (*P* < 0.05) were located within the RBD for HCoV-229E, 60.5 per cent (*P* < 0.01) for HCoV-OC43, and 86.7 per cent (*P* = 0.08) for IAV H3N2 (Table 2).

**Table 3.** Likelihood ratio test for positive selection in viral glycoprotein genes.

Virus	lnL_0_	lnL_1_	2ΔlnL	df	*P* value
HCoV-229E	−7293.1	−7288.4	9.4	2	9.1 × 10^-3^
HCoV-OC43	−10513.1	−10474.5	77.1	2	<1.1 × 10^-16^
IAV H3N2	−10040.9	−10032.4	17.1	2	1.9 × 10^-4^

lnL, log likelihoods estimated using PAML; lnL_0,_ estimated under M7; lnL_1,_ estimated under M8, 2ΔlnL, 2(lnL_1_ - lnL_0_); df, degrees of freedom for chi-square test.

Selection pressure analyses can be biased by genetic changes that are not considered within the framework of the programs designed to detect and differentiate change resulting from virus–host population-level interactions. Within SARS-CoV-2 and to a lesser extent in other HCoVs, host-mediated editing of the viral genome can lead to erroneous assumption of sites evolving under pressure, such as mutations by the apolipoprotein B mRNA-editing enzyme, catalytic polypeptide-like (APOBEC) family leading to C→U transitions (Simmonds 2020). Therefore, we determined if the sites under positive selection were C→U transitions that may be suggestive of APOBEC-mediated editing (Simmonds 2020). We found that 1/15 (6.7%) of these sites had a C→U transition in HCoV-229E, 4/43 (9.3%) in HCoV-OC43, whereas no such transition was found in IAV H3N2 (Supplementary Tables S4–S6), implying that most sites showing evidence for positive selection were not generated by APOBEC-like editing.

In sum, sites under positive selection within the RBD of HCoV may be particularly relevant for immune escape and antigenic drift, as has been reported for IAV H3N2 (Koel et al. 2013; Raymond et al. 2018).

## 4. Discussion

We evaluated the evolutionary dynamics of two ubiquitous endemic HCoV in comparison to IAV H3N2.

We found several similarities between both HCoV and IAV H3N2, including tree shape and the location of both non-synonymous mutations and sites under positive selection. Genetic variability potentially compatible with antigenic drift has been described in preliminary studies analyzing the S genes of HCoV-229E (Chibo and Birch 2006) and HCoV-OC43 (Ren et al. 2015) individually. Our analysis of relatively larger HCoV and IAV datasets using identical methodology confirmed those preliminary studies and allowed direct comparisons between the viruses under study. Our data demonstrate considerably lower gene-wide change over time in HCoV than in IAV, which may imply a prolonged ability of vaccine-induced immune responses to neutralize coronavirus variants arising over time.

However, even a single amino acid exchange can dramatically affect immune escape, as demonstrated for many viruses infecting humans. In IAV, the recently emerged HA mutation K166Q reduced HA inhibition titers by ≥two-fold (Linderman et al. 2014), prompting for modification of the H1N1 vaccine strain in 2017 (Raymond et al. 2018). In Polioviruses, immune escape mutations were associated with an outbreak of poliomyelitis in the Republic of Congo in 2010 (Drexler et al. 2014). Even in hepatitis B virus that evolves several orders of magnitude slower than IAV and Polioviruses (Muhlemann et al. 2018), vaccine breakthrough after mother-to-child transmission and subsequent immunization of the neonate was linked to a single mutation in the glycoprotein (Romano et al. 2015). It is therefore not unlikely that single amino acid changes can have a dramatic impact on HCoV antigenicity. Indeed, differential neutralization of HCoV-229E strains was linked to substitutions within the S1 receptor-binding loops within the RBD (Shirato et al. 2012; Wong et al. 2017). Moreover, it was recently demonstrated that historical human sera collected from 1985 to 1990 had lower neutralizing activity to pseudotyped viruses bearing the S of HCoV-229E strains isolated eight to seventeen years later (Eguia et al. 2020), suggesting antigenic drift.

One year after SARS-CoV-2 was first reported in humans, several mutations in S leading to deletions or amino acid exchanges have emerged independently in several countries (e.g. UK, South Africa, and Brazil) and are becoming regionally predominant (Plante et al. 2020; Tegally et al. 2020; Faria et al. 2021; Volz et al. 2021). These amino acid exchanges or deletions in S can lead to increased transmission by increasing infectivity (e.g. D614G) (Plante et al. 2020), enhancing human ACE2-binding affinity (e.g. N439K and N501Y) (Starr et al. 2020; Thomson et al. 2020), or conferring partial immune escape by reduction of neutralizing activity to both human-derived polyclonal sera and monoclonal antibodies (e.g. N439K, E484K, K417N, N501Y, Δ69/70) (Kemp et al. 2021; Thomson et al. 2021; Weisblum et al. 2020; McCarthy et al. 2021; Wang et al. 2021). Most of these mutations are located within the RBD, which is indicative of the relevance of that genomic domain for viral adaptive evolution and consistent with our results and those of other studies (Wong et al. 2017; Weisblum et al. 2020). Some of the immune escape mutations were reported to emerge in immunocompromised individuals after treatment with monoclonal antibodies and convalescent plasma (Choi et al. 2020; Kemp et al. 2021). Although it is possible that prolonged within-host evolution of SARS-CoV-2 in immunocompromised individuals can enhance the emergence of mutations conferring immune escape, intense uncontrolled community transmission of SARS-CoV-2 will facilitate the emergence of escape variants irrespective of host immune status. Immune escape is all the more worrying because weak immune responses against SARS-CoV-2 have been reported to occur particularly in mild and asymptomatic infections (Okba et al. 2020; Wajnberg et al. 2020) and sporadically linked to re-infection with SARS-CoV-2 (Gupta et al. 2020; To et al. 2020; Tillett et al. 2021). Finally, neutralization assays of viruses pseudotyped with SARS-CoV-2 spike variants (e.g. UK-B.1.1.7 and South Africa-B.1.351) demonstrated reduced levels of neutralization by vaccinee-derived antisera (Madhi et al. 2021; Tada et al. 2021; Wu et al. 2021). COVID-19 vaccines may therefore require constant evaluation during pandemic SARS-CoV-2 spread.

Limitations in our study include the small number of sequences per year and the different dataset sizes. Another limitation was the use of only S gene sequences, as T-cell reactivity has been reported for other SARS-CoV-2 proteins such as M, N and several non-structural proteins (Grifoni et al. 2020; Le Bert et al. 2020). However, the S protein is the main target of neutralizing antibodies (Premkumar et al. 2020), and therefore the main viral protein used for vaccine development (Jackson et al. 2020; Mulligan et al. 2020). 

Alike SARS-CoV-2, IAV H3N2 emerged relatively recently in 1968 from an animal reservoir (Smith et al., 2009). It seems plausible that the evolutionary trajectory of SARS-CoV-2 will bear similarities with that of IAV H3N2 during the pandemic phase and in the immediate aftermath, characterized by viral adaptation and accumulation of mutations in the RBD. Under this assumption, it seems plausible that the efficacy of COVID-19 vaccines against emerging SARS-CoV-2 variants requires careful validation and regular vaccine update during pandemic spread. In contrast, seasonal HCoV emergence likely dates back longer time spans, potentially implying several hundred years of purifying selection (Vijgen et al. 2006; Pfefferle et al. 2009; Corman et al. 2015) that limit comparability of HCoV evolution with pandemic SARS-CoV-2 evolution during intense transmission facilitated by global connectivity (Findlater and Bogoch 2018). Nonetheless, the unique presence of the highly conserved proofreading protein nsp14 across all coronaviruses implies that SARS-CoV-2 evolution will bear similarities with seasonal HCoV evolution in a post-pandemic scenario. Enhanced stability of COVID-19 vaccines in the post-pandemic stage can thus be expected compared to influenza vaccines, both due to viral properties and due to relatively stronger T-cell responses afforded by most COVID-19 vaccines (Corbett et al. 2020; Sahin et al. 2020) compared to current influenza vaccines (Kang et al. 2004). 

## Supplementary data

Supplementary data are available at *Virus Evolution* online.

## Supplementary Material

veab020_Supplementary_Data

## Data Availability

GenBank accession numbers of all sequences used in final datasets are shown in Table S1. **Conflict of interest:** None declared.
