# Supplementary material for: The evolutionary dynamics of endemic human coronaviruses
Source: Virus Evol. 2021 Mar 20;7(1):veab020. doi: 10.1093/ve/veab020 (PMC7980080; doi:10.1093/ve/veab020)
Supplement: veab020_Supplementary_Data [file veab020_supplementary_data.zip › Table S2-S6.pdf]

Table S2. Tree shape statistics based in Bayesian trees using subsampled datasets

Tree statistic	Description	HCoV-229E	HCoV-OC43	IAV H3N2
Colless index#	Assess overall asymmetry	0.74	0.35	0.36
Sackin index#	Assess overall asymmetry	0.81	0.46	0.40
Cherry number	Count branches with two tips	0.44	0.55	0.55
Number internal nodes	Count internal nodes with a single tip child	0.6	0.47	0.46
Ladder length	Measures mean size of ladders	3.5	2.14	2.85
Staircase-ness#	Count proportion of imbalanced subtrees	0.77	0.72	0.69

Value of 1 indicates perfect asymmetry, value of 0 indicates perfect symmetry (Colijn and Gardy, 2014)

Table S3. Evolutionary rates using different clock models

Clock model	HCoV-229E (95% HPD)	HCoV-OC43 (95% HPD)	IAV H3N2 (95% HPD)	Fold of change (H3N2/229E)	Fold of change (H3N2/OC43)
**Strict**	6.5×10^{-4} (5.4–7.5×10^{-4})	$5.7x10^{-4}$ (5–6.5×10^{-4})	2.5×10^{-3} (2.3–2.7×10^{-3})	3.85	4.39
**Relaxed log normal**	7.4×10^{-4} (5.7–9.2×10^{-4})	$6.5x10^{-4}$ (5.3–7.8×10^{-4})	2.7×10^{-3} (2.5–2.9×10^{-3})	3.65	4.15

Table S4. Amino acid residues in HCoV-229E Spike coding frame under positive selection

Site	Methods	SLAC	Fubar	MEME	PAML	Mutation*
22	1	n.s.	0.94	n.s.	n.s.	T → C
41	1	n.s.	0.941	n.s.	n.s.	C → A/T
89	1	n.s.	0.959	n.s.	n.s.	G → A
90	1	n.s.	0.938	n.s.	n.s.	T → C
104	1	n.s.	0.984	n.s.	n.s.	A → C
125	1	n.s.	n.s.	0.01	n.s.	GA → CG
175	1	n.s.	0.908	n.s.	n.s.	G → A
313	2	n.s.	0.936	0.06	n.s.	CC → GT
315	2	n.s.	0.962	n.s.	0.991	G → A
324	1	n.s.	0.95	n.s.	n.s.	G → A
355	2	n.s.	0.963	n.s.	0.972	G → T → C
356	1	n.s.	0.948	n.s.	n.s.	G → A → C
405	1	n.s.	0.937	n.s.	n.s.	AT → CA → TA
453	2	n.s.	0.942	0.01	n.s.	T → G
790	1	n.s.	0.93	n.s.	n.s.	A → G

Statistical criteria: SLAC and MEME $p<0.1$, FUBAR and PAML (M8 BEB) PPr>0.9 and $p<0.05$; not significant, n.s.; Receptor binding domain sites in gray, S1 and S2 sites separated by double line; *Mutations were annotated by order of appearance, lineages were not taken into account

Table S5. Amino acid residues in HCoV-OC43 Spike coding frame under positive selection

Site	Methods	SLAC	Fubar	MEME	PAML	Mutation*
26	2	n.s.	0.978	n.s.	0.996	T → G
27	3	n.s.	0.973	0.05	0.996	TA → CT
33	1	n.s.	0.935	n.s.	n.s.	G → A
34	1	n.s.	n.s.	n.s.	0.963	GTT → AAA → AGA
**38**	**4**	**0.008**	**0.995**	**0.03**	**1**	**C → T**
**40**	**2**	**n.s.**	**0.959**	**n.s.**	**0.979**	**C → T**
43	1	n.s.	0.909	n.s.	n.s.	C → T - C→ A - A → T
90	2	n.s.		0	0.999	CTG → AAA → TTA/CTA
93	1	n.s.	n.s.	0.09	n.s.	C → A
120	2	n.s.	0.974	0.01	n.s.	G → A/C - T → A/G
152	1	n.s.	0.944	n.s.	n.s.	T ←→ C
153	2	n.s.		0.07	0.983	GAT → ACA
176	1	n.s.	n.s.	0.07	n.s.	T → C
**185**	**1**	**n.s.**	**n.s.**	**n.s.**	**0.97**	**C → T**
195	4	0.065	0.985	0	0.991	T → C/A - G → T
199	2	n.s.	0.923	0.07	n.s.	T → C
215	3	n.s.	0.973	0.02	0.975	G → A - A → C
263	1	n.s.	0.918	n.s.	n.s.	T → A - A → C
265	4	0.039	0.987	0.04	1	G → T/C
266	2	n.s.	0.952	0.06	n.s.	G → A - T → A
267	1	n.s.	n.s.	0.08	n.s.	GAT → ATT/GAC
271	1	n.s.	n.s.	0.06	n.s.	T → C
413	2	n.s.	0.941	n.s.	0.992	G → C
425	1	n.s.	n.s.	0.08	n.s.	T → A
474	2	n.s.	n.s.	0.01	0.992	AA → GT
485	2	n.s.	0.958	0.06	n.s.	GA → AG
503	2	n.s.	0.948	0	n.s.	AAA → AGT
524	1	n.s.	n.s.	n.s.	1	A → C
537	1	n.s.	n.s.	0.09	n.s.	C → G
538	2	n.s.	0.954	n.s.	0.98	T → C
542	1	n.s.	0.946	n.s.	n.s.	G → A
570	1	n.s.	0.947	n.s.	n.s.	G → A
571	1	n.s.	n.s.	0.04	n.s.	G → A
624	1	n.s.	n.s.	n.s.	0.995	ATT → GAA
757	1	n.s.	0.96	n.s.	n.s.	G → T
**759**	**1**	**n.s.**	**0.941**	**n.s.**	**n.s.**	**C → T**
766	3	n.s.	0.96	0.03	0.995	G → A
992	1	n.s.	n.s.	0	n.s.	T ←→ A
1024	1	n.s.	0.946	n.s.	n.s.	G → T/C
1048	2	n.s.	0.968	n.s.	0.969	G → T
1209	1	n.s.	0.908	n.s.	n.s.	C → A
1258	1	n.s.	0.962	n.s.	n.s.	G → C - A → T
1353	3	0.095	0.975	0.01	0.988	A → T

Statistical criteria: SLAC and MEME p<0.1, FUBAR and PAML (M8 BEB) PPr>0.9 and p<0.05; not significant, n.s.; Receptor binding domain sites in gray, S1 and S2 sites separated by double line; C→U transition highlighted; *Mutations were annotated by order of appearance, lineages were not taken into account

Table S6. Amino acid residues in IAV-H3N2 HA coding frame under positive selection

Site	Methods	SLAC	Fubar	MEME	PAML	Mutation*
49	2	0.093	n.s.	0.06	n.s.	A → G
63	1	n.s.	n.s.	0.09	n.s.	T → C
66	2	0.092	n.s.	0.08	n.s.	A → G
70	1	n.s.	n.s.	0.01	n.s.	A → G – G → T
147	1	n.s.	n.s.	0.06	n.s.	G → A - GCT → AAA
151	3	0.015	0.975	0	n.s.	GAC → ACA
158	1	0.064	n.s.	n.s.	n.s.	G → A
160	4	0.031	0.977	0.02	0.957	G → A - T → A/G
171	1	n.s.	n.s.	0.06	n.s.	C → A - A → C
175	1	n.s.	n.s.	0.09	n.s.	A → T/C
189	1	n.s.	n.s.	0.1	n.s.	A → C/G
209	1	n.s.	n.s.	0.01	n.s.	A → T - G → T/C
215	1	n.s.	n.s.	0.05	n.s.	T → C/A/G
242	1	n.s.	n.s.	n.s.	0.980	A → G - A → T
320	1	n.s.	n.s.	0.03	n.s.	A → C/G
454	1	n.s.	n.s.	0	n.s.	G → C - T → C
545	3	0.090	0.902	0.06	n.s.	G → A/T

Statistical criteria: SLAC and MEME p<0.1, FUBAR and PAML (M8 BEB) PPr>0.9 and p<0.05; not significant, n.s.; Receptor binding domain sites in gray, HA1 and HA2 sites separated by double line; *Mutations were annotated by order of appearance, lineages were not taken into account
