# Supplementary material for: The evolutionary dynamics of endemic human coronaviruses
Source: Virus Evol. 2021 Mar 20;7(1):veab020. doi: 10.1093/ve/veab020 (PMC7980080; doi:10.1093/ve/veab020)
Supplement: veab020_Supplementary_Data [file veab020_supplementary_data.zip › TableS1.pdf]

Table S1. GenBank accession numbers of spike and hemagglutinin complete gene sequences in final datasets

Virus	Accession no.	Strain	Country	Year
HCoV-229E	DQ243964[&]	-	Australia	1979
HCoV-229E	DQ243965	-	Australia	1982
HCoV-229E	DQ243966	-	Australia	1982
HCoV-229E	DQ243968	-	Australia	1982
HCoV-229E	DQ243969	-	Australia	1982
HCoV-229E	DQ243970[&]	-	Australia	1982
HCoV-229E	DQ243971[&]	-	Australia	1984
HCoV-229E	DQ243972	-	Australia	1984
HCoV-229E	KF514429[&]	892-11	USA	1989
HCoV-229E	DQ243973[&]	-	Australia	1990
HCoV-229E	DQ243974[&]	-	Australia	1992
HCoV-229E	DQ243975	-	Australia	1992
HCoV-229E	DQ243976	-	Australia	1992
HCoV-229E	KF514430[&]	933-50	USA	1993
HCoV-229E	KF514433	933-40	USA	1993
HCoV-229E	KF514432	932-72	USA	1993
HCoV-229E	KF514431[&]	953-49	USA	1995
HCoV-229E	DQ243977[&]	-	Australia	2001
HCoV-229E	DQ243978	-	Australia	2001
HCoV-229E	DQ243979[&]	-	Australia	2002
HCoV-229E	DQ243980[&]	-	Australia	2003
HCoV-229E	DQ243983	-	Australia	2003
HCoV-229E	DQ243984	-	Australia	2003
HCoV-229E	DQ243985	-	Australia	2003
HCoV-229E	DQ243986	-	Australia	2003
HCoV-229E	AB691764[&]	1121	Japan	2004
HCoV-229E	AB691765	826	Japani	2004
HCoV-229E	AB691766	1948	Japan	2004
HCoV-229E	KM055557[&]	748	China	2005
HCoV-229E	KM055559	857	China	2005
HCoV-229E	KM055551[&]	359A	China	2007
HCoV-229E	KM055554	494A	China	2008
HCoV-229E	KM055555	507A	China	2008
HCoV-229E	KM055556[&]	693A	China	2008
HCoV-229E	AB691767[&]	1	Japan	2008
HCoV-229E	KM055533[&]	1518A	China	2009
HCoV-229E	KM055534	1546A	China	2009
HCoV-229E	KM055535	1649A	China	2009
HCoV-229E	KM055536	1658A	China	2009
HCoV-229E	KM055545	8339	China	2009

HCoV-229E	KM055547	8425	China	2009
HCoV-229E	KM055553	1482A	China	2009
HCoV-229E	KM055558	8348	China	2009
HCoV-229E	KM055560	1424A	China	2009
HCoV-229E	JX503060[&]	349	Netherlands	2010
HCoV-229E	KM055538[&]	1761A	China	2010
HCoV-229E	KM055541	1827A	China	2010
HCoV-229E	KM055543	1990A	China	2010
HCoV-229E	KM055544	2764A	China	2011
HCoV-229E	KM055531[&]	11949	China	2011
HCoV-229E	KM055548	2861A	China	2011
HCoV-229E	KM055549	2961A	China	2011
HCoV-229E	KM055550	2981A	China	2011
HCoV-229E	MH048989[&]	Lille	France	2014
HCoV-229E	KY967357[&]	SC2872	USA	2015
HCoV-229E	KY983587	SC3112	USA	2015
HCoV-229E	KY621348[&]	SC379	USA	2016
HCoV-229E	MN369046[&]	SC9724	USA	2018
HCoV-229E	MN306046[&]	SC0865	USA	2019
HCoV-229E	JX503061	J0304	Italy	2009
HCoV-229E	KY996417	UF-1	USA	2016
HCoV-229E	MF542265[&]	Haiti-1	Haiti	2016
HCoV-229E	AF344187[†]	P11A		2001
HCoV-229E	AF344188[†]	P11B		2001
HCoV-229E	AF344189[†]	P100E		2001
HCoV-229E	DQ243963[†]	ATCC	VR-740	1973
HCoV-229E	AF344186[†]	RWStock		2001
HCoV-OC43	KF530093[#&]	832-27	USA	1983
HCoV-OC43	KF530060[&]	851-15	USA	1985
HCoV-OC43	KF530083	873-19	USA	1987
HCoV-OC43	KF530087	873-6	USA	1987
HCoV-OC43	KF530077[&]	873-16	USA	1987
HCoV-OC43	KF530085[#]	871-25	USA	1987
HCoV-OC43	KF530073[&]	8912-37	USA	1989
HCoV-OC43	KF530066	901-33	USA	1990
HCoV-OC43	KF530065	901-41	USA	1990
HCoV-OC43	KF530061[&]	901-43	USA	1990
HCoV-OC43	KF530088	901-54	USA	1990
HCoV-OC43	KF530095	912-6	USA	1991
HCoV-OC43	KF530082	912-11	USA	1991
HCoV-OC43	KF530091	911-58	USA	1991
HCoV-OC43	KF530076	911-11	USA	1991
HCoV-OC43	KF530067[&]	912-10	USA	1991
HCoV-OC43	KF530079	913-29	USA	1991

HCoV-OC43	KF530089	911-66	USA	1991
HCoV-OC43	KF530074	9212-33	USA	1992
HCoV-OC43	KF530071[&]	925-1	USA	1992
HCoV-OC43	KF530084	951-18	USA	1995
HCoV-OC43	KF530062	952-23	USA	1995
HCoV-OC43	KF530075	953-23	USA	1995
HCoV-OC43	KF530059[&]	951-15	USA	1995
HCoV-OC43	KF530098	965-6	USA	1996
HCoV-OC43	KF530078	9612-29	USA	1996
HCoV-OC43	KF530064	9612-9	USA	1996
HCoV-OC43	KF530063[&]	9612-48	USA	1996
HCoV-OC43	KF530072[&]	9712-13	USA	1997
HCoV-OC43	KF530080	9712-31	USA	1997
HCoV-OC43	KF530099	971-5	USA	1997
HCoV-OC43	KF530069[&]	982-4	USA	1998
HCoV-OC43	KF530081	991-5	USA	1999
HCoV-OC43	KF530070[&]	991-19	USA	1999
HCoV-OC43	KF530068[&]	007-11	USA	2000
HCoV-OC43	KF530092[#]	008-5	USA	2000
HCoV-OC43	KF963231	Caen02	France	2001
HCoV-OC43	KF963230[#&]	Caen01	France	2001
HCoV-OC43	KF963233[#]	Caen04	France	2002
HCoV-OC43	KF963232[&]	Caen03	France	2002
HCoV-OC43	KY014281[#]	4	France	2002
HCoV-OC43	AY903460	19572	Belgium	2004
HCoV-OC43	AY903458	36638	Belgium	2004
HCoV-OC43	AY903455[&]	34364	Belgium	2004
HCoV-OC43	JN129835	HK04-02	HongKong	2004
HCoV-OC43	JN129834[&]	HK04-01	HongKong	2004
HCoV-OC43	KF572816[#&]	22905	China	2005
HCoV-OC43	KF963236[#&]	Caen07	France	2005
HCoV-OC43	KF572818	364706	China	2006
HCoV-OC43	KF572817[&]	358206	China	2006
HCoV-OC43	KF572843	495407	China	2007
HCoV-OC43	KF572844	524007	China	2007
HCoV-OC43	KF572845	533107	China	2007
HCoV-OC43	KF572846	534507	China	2007
HCoV-OC43	KF572850	544207	China	2007
HCoV-OC43	KF572851	544507	China	2007
HCoV-OC43	KF572853	547907	China	2007
HCoV-OC43	KF572854	548407	China	2007
HCoV-OC43	KF572855	548507	China	2007
HCoV-OC43	KF572857	551707	China	2007
HCoV-OC43	KF572859	556607	China	2007

HCoV-OC43	KF572862	562507	China	2007
HCoV-OC43	KF572864	69A07	China	2007
HCoV-OC43	KF572852[#]	547207	China	2007
HCoV-OC43	KF572858[#]	551907	China	2007
HCoV-OC43	KF572820[#]	079A07	China	2007
HCoV-OC43	KF572842[#&]	479507	China	2007
HCoV-OC43	KF572860[#]	559507	China	2007
HCoV-OC43	KF572863[#]	565607	China	2007
HCoV-OC43	KF572847[#]	535207	China	2007
HCoV-OC43	KF572848[#]	537007	China	2007
HCoV-OC43	KF572849[#]	541407	China	2007
HCoV-OC43	KF572856[#]	550807	China	2007
HCoV-OC43	KF572819[#]	039A07	China	2007
HCoV-OC43	KF572828	1157A08	China	2008
HCoV-OC43	KF572824[&]	1034A08	China	2008
HCoV-OC43	KF572827	1135A08	China	2008
HCoV-OC43	KF572872	978A08	China	2008
HCoV-OC43	KF572868	892A08	China	2008
HCoV-OC43	KF572869	894209	China	2009
HCoV-OC43	KF572815[&]	1489A09	China	2009
HCoV-OC43	KF572865	809909	China	2009
HCoV-OC43	KF572870	900109	China	2009
HCoV-OC43	KF572837	1593A09	China	2009
HCoV-OC43	KF572836	1591A09	China	2009
HCoV-OC43	KF572866	816409	China	2009
HCoV-OC43	KF572835	1382A09	China	2009
HCoV-OC43	KF572867	837509	China	2009
HCoV-OC43	KF963240[&]	Caen11	France	2009
HCoV-OC43	KF572838	2134A10	China	2010
HCoV-OC43	KF572825	1057410	China	2010
HCoV-OC43	KF572823	1029010	China	2010
HCoV-OC43	KF572839	2151A10	China	2010
HCoV-OC43	KF572821[&]	1010810	China	2010
HCoV-OC43	KF572829[&]	1193011	China	2011
HCoV-OC43	KJ958218	LY341	China	2011
HCoV-OC43	KX344031[&]	LRTI238	Mexico	2011
HCoV-OC43	KF963242[&]	Caen13	France	2011
HCoV-OC43	KJ958219	LY342	China	2011
HCoV-OC43	KX538968	MY-U464	Malaysia	2012
HCoV-OC43	KX538976	MY-U1057	Malaysia	2012
HCoV-OC43	KX538970	MY-U710	Malaysia	2012
HCoV-OC43	KX538972	MY-U774	Malaysia	2012
HCoV-OC43	KX538966	MY-U236	Malaysia	2012
HCoV-OC43	KF963243[&]	Caen14	France	2012

HCoV-OC43	KF572831[&]	1268912	China	2012
HCoV-OC43	KF572832	1269112	China	2012
HCoV-OC43	KF572841	3269A12	China	2012
HCoV-OC43	KX538964[&]	MYU002	Malaysia	2012
HCoV-OC43	KX538974	MY-U945	Malaysia	2012
HCoV-OC43	KX538975	MY-U1024	Malaysia	2012
HCoV-OC43	KF572833	1269412	China	2012
HCoV-OC43	KX538965	MY-U208	Malaysia	2012
HCoV-OC43	KX538977	MY-U1140	Malaysia	2012
HCoV-OC43	KX538967	MY-U413	Malaysia	2012
HCoV-OC43	KF572840	3098A12	China	2012
HCoV-OC43	KU745537[&]	3791A	China	2013
HCoV-OC43	KX538979	MY-U1975	Malaysia	2013
HCoV-OC43	KX538978[&]	MY-U1758	Malaysia	2013
HCoV-OC43	MK303621	MDS4	France	2013
HCoV-OC43	KF963244[&]	Caen15	France	2013
HCoV-OC43	MK303624	MDS14	France	2013
HCoV-OC43	LC315646[&]	SGH-36	Japan	2014
HCoV-OC43	KU745534	13969	China	2014
HCoV-OC43	KU745547	HB14018d7	China	2014
HCoV-OC43	KU745535	14007	China	2014
HCoV-OC43	KU745539	4086A	China	2014
HCoV-OC43	KU745548	SZ14014d3	China	2014
HCoV-OC43	KU745536	14012	China	2014
HCoV-OC43	MK303622	MDS11	France	2014
HCoV-OC43	MK303623	MDS12	France	2014
HCoV-OC43	MK303620[&]	MDS2	France	2014
HCoV-OC43	KU745533[&]	13963	China	2014
HCoV-OC43	KU745538	4068A	China	2014
HCoV-OC43	MG197710	BJ-124	China	2015
HCoV-OC43	MG197713	BJ-221	China	2015
HCoV-OC43	MG197715	GZYF-26	China	2015
HCoV-OC43	MG197716	WZ-303	China	2015
HCoV-OC43	MG197717	WZ-522	China	2015
HCoV-OC43	MG197722	YC-207	China	2015
HCoV-OC43	LC315648[&]	SGH-06	Japan	2015
HCoV-OC43	KU745544	4450A	China	2015
HCoV-OC43	KU745543[&]	4449A	China	2015
HCoV-OC43	KU745545	4452A	China	2015
HCoV-OC43	KY967358	SC2770	USA	2015
HCoV-OC43	KY967356	SC2924	USA	2015
HCoV-OC43	KY967359	SC2730	USA	2015
HCoV-OC43	KY983585[&]	SC2854	USA	2015
HCoV-OC43	KY967361	SC2345	USA	2015

HCoV-OC43	MK303619[&]	MDS6	France	2015
HCoV-OC43	KY369906	SC622	USA	2016
HCoV-OC43	KY554972[#]	N07-1541B433X	USA	2016
HCoV-OC43	KY554974[#]	N08-33B360X	USA	2016
HCoV-OC43	MF374983	TCNP0070	USA	2016
HCoV-OC43	KY684759	SC2269	USA	2016
HCoV-OC43	MG197723[&]	HZ-459	China	2016
HCoV-OC43	MH121121	ACRI0213	USA	2016
HCoV-OC43	KY369905[&]	SC831	USA	2016
HCoV-OC43	KY674920[#]	N09-595B	USA	2016
HCoV-OC43	KY554975[#]	N09-382B	USA	2016
HCoV-OC43	KY369907	SC9741	USA	2016
HCoV-OC43	MG977451	TNP12636	IvoryCoast	2016
HCoV-OC43	MG977445[&]	TNPF17902	IvoryCoast	2016
HCoV-OC43	MG977444[&]	TNPF1778	IvoryCoast	2017
HCoV-OC43	MF374985[&]	TCNP00212	USA	2017
HCoV-OC43	MN306042	SC0839	USA	2019
HCoV-OC43	MN310478	SC0776	USA	2019
HCoV-OC43	MN306053	SC9430	USA	2019
HCoV-OC43	MN306041[&]	SC0810	USA	2019
HCoV-OC43	KF572809[§]	2058A	China	2010
HCoV-OC43	AY903459[§]	87309	Belgium	2003
HCoV-OC43	AY903457[§]	37767	Belgium	2003
HCoV-OC43	AY903454[§]	89996	Belgium	2003
HCoV-OC43	AY903456[§]	84020	Belgium	2003
HCoV-OC43	KF572804[§]	1783A	China	2010
HCoV-OC43	KF572805[§]	1908A10	China	2010
HCoV-OC43	KF572806[§]	1919A10	China	2010
HCoV-OC43	KF572807[§]	192606	China	2006
HCoV-OC43	KF572808[§]	1997A10	China	2010
HCoV-OC43	KF572810[§]	2145A10	China	2010
HCoV-OC43	KF572811[§]	2941A11	China	2011
HCoV-OC43	KF572812[§]	3074A12	China	2012
HCoV-OC43	KF572813[§]	3184A12	China	2012
HCoV-OC43	KF572814[§]	3194A12	China	2012
HCoV-OC43	KF963234[§]	Caen05	France	2003
HCoV-OC43	KF963237[§]	Caen08	France	2006
HCoV-OC43	KF963238[§]	Caen09	France	2007
HCoV-OC43	KF963239[§]	Caen10	France	2008
HCoV-OC43	KF963241[§]	Caen12	France	2010
HCoV-OC43	KU745540[§]	4400A	China	2015
HCoV-OC43	KU745541[§]	4436A	China	2015
HCoV-OC43	KU745546[§]	4467A	China	2015

HCoV-OC43	KU131570[§]	London	UK	2011
HCoV-OC43	KY014282[§]	9	France	2007
HCoV-OC43	KY967360[§]	SC2476	USA	2015
HCoV-OC43	MF314143[§]	ACRI0052	USA	2016
HCoV-OC43	MG197709[§]	BJ-112	China	2015
HCoV-OC43	MG197711[§]	BJ-164	China	2015
HCoV-OC43	MG197714[§]	CC-23	China	2015
HCoV-OC43	MG197718[§]	YC-55	China	2015
HCoV-OC43	MG197719[§]	YC-67	China	2015
HCoV-OC43	MG197720[§]	YC-68	China	2015
HCoV-OC43	MG197721[§]	YC-72	China	2015
HCoV-OC43	MK303625[§]	MDS16	France	2015
HCoV-OC43	MK327281[§]	MDS15	France	2014
HCoV-OC43	MN306043[§]	SC0841	USA	2019
HCoV-OC43	MN310476[§]	SC9428	USA	2019
HCoV-OC43	KU745542[§]	4446A	China	2015
HCoV-OC43	KF572822[§]	1028510	China	2010
HCoV-OC43	KF963229[†]	VR759	France	1967
HCoV-OC43	AY391777[†]	ATCCVR-759		1967
HCoV-OC43	AY585229[†]	France	France	2001
HCoV-HKU1	AY597011.2	HKU1A	Hong Kong	2004
HCoV-HKU1	DQ339101	N5P8AB	Hong Kong	2005
HCoV-HKU1	DQ415902	N25B	Hong Kong	2005
HCoV-HKU1	DQ415903	N3A	Hong Kong	2003
HCoV-HKU1	DQ437607	N11A	Hong Kong	2004
HCoV-HKU1	DQ437608	N13A	Hong Kong	2004
HCoV-HKU1	DQ437609	N14A	Hong Kong	2004
HCoV-HKU1	DQ437610	N15B	Hong Kong	2004
HCoV-HKU1	DQ437611	N16C	Hong Kong	2004
HCoV-HKU1	DQ437614	N19A	Hong Kong	2004
HCoV-HKU1	DQ437616	N21C	Hong Kong	2004
HCoV-HKU1	DQ437617	N22C	Hong Kong	2005
HCoV-HKU1	HM034837	Caen1	France	2005
HCoV-HKU1	KF430197	HTBEClab22	Brazil	2007
HCoV-HKU1	KF430198	HTBEClab21	Brazil	2006
HCoV-HKU1	KF430201	18	USA	2010
HCoV-HKU1	KF430203	HTBEClab23	Brazil	2006
HCoV-HKU1	KF686343	13	USA	2010
HCoV-HKU1	KF686344	15	USA	2009
HCoV-HKU1	KT779555	BJ01-p3	China	2009
HCoV-HKU1	KT779556	BJ01-p9	China	2009
HCoV-HKU1	KY674921	N08-87	USA	2016
HCoV-HKU1	KY674943	N09-1605B	USA	2016
HCoV-HKU1	KY983584	SC2628	USA	2015

HCoV-HKU1	LC315650	SGH-15	Japan	2014
HCoV-HKU1	LC315651	SGH-18	Japan	2016
HCoV-HKU1	MH940245	SI17244	Thailand	2017
HCoV-HKU1	MK167038§	SC2521	USA	2017
HCoV-NL63	KF530110	838-9	USA	1983
HCoV-NL63	KF530106	8712-17	USA	1987
HCoV-NL63	AY518894	8Mo	Netherlands	1988
HCoV-NL63	KF530114	891-4	USA	1989
HCoV-NL63	KF530108	891-6	USA	1989
HCoV-NL63	KF530109	903-28	USA	1990
HCoV-NL63	KF530113	905-25	USA	1990
HCoV-NL63	KF530111	901-24	USA	1990
HCoV-NL63	KF530104	904-20	USA	1990
HCoV-NL63	KF530107	911-56	USA	1991
HCoV-NL63	KF530105	012-31	USA	2001
HCoV-NL63	AY567487	NetherlandsI	Netherlands	2003
HCoV-NL63	JQ765568	193	USA	2005
HCoV-NL63	JQ765571	271	USA	2005
HCoV-NL63	JQ765572	347	USA	2005
HCoV-NL63	JQ765573	1062	USA	2005
HCoV-NL63	JQ765574	1862	USA	2005
HCoV-NL63	JQ765575	1876	USA	2005
HCoV-NL63	KM055638	180A	China	2007
HCoV-NL63	KM055639	197A	China	2007
HCoV-NL63	KM055640	225A	China	2007
HCoV-NL63	KM055642	277A	China	2007
HCoV-NL63	KM055644	374A	China	2007
HCoV-NL63	KM055647	188A	China	2007
HCoV-NL63	KM055648	954A	China	2008
HCoV-NL63	KM055649	996A	China	2008
HCoV-NL63	KM055650	930A	China	2008
HCoV-NL63	KM055634	1023A	China	2008
HCoV-NL63	KM055636	940A	China	2008
HCoV-NL63	KM055637	945A	China	2008
HCoV-NL63	KM055632	1014A	China	2008
HCoV-NL63	JQ765566	16	USA	2008
HCoV-NL63	JX104161	CBJ037	China	2008
HCoV-NL63	JQ765564	14	USA	2009
HCoV-NL63	JQ765565	15	USA	2009
HCoV-NL63	JQ765567	20	USA	2009
HCoV-NL63	JX524171	CBJ123	China	2009
HCoV-NL63	KM055633	2093A	China	2010
HCoV-NL63	JQ771056	25	USA	2010
HCoV-NL63	JQ771057	28	USA	2010

HCoV-NL63	JQ771058	31	USA	2010
HCoV-NL63	JQ771059	35	USA	2010
HCoV-NL63	JQ771060	36	USA	2010
HCoV-NL63	MG428699	KilifiHH5709	Kenya	2010
HCoV-NL63	MG428700	KilifiHH1602	Kenya	2010
HCoV-NL63	MG428701	KilifiHH0512	Kenya	2010
HCoV-NL63	MG428702	KilifiHH3807	Kenya	2010
HCoV-NL63	MG428703	KilifiHH0511	Kenya	2010
HCoV-NL63	MG428704	KilifiHH5402	Kenya	2010
HCoV-NL63	MG428705	KilifiHH0522	Kenya	2010
HCoV-NL63	MG428706	KilifiHH3808	Kenya	2010
HCoV-NL63	MG772808	CN0601	South Korea	2014
HCoV-NL63	KX179494	Haiti2	Haiti	2015
HCoV-NL63	KX179495	Haiti3	Haiti	2015
HCoV-NL63	KY554967	N06-1144B	USA	2016
HCoV-NL63	KY554968	N07-185B	USA	2016
HCoV-NL63	KY554970	N07-324B182X	USA	2016
HCoV-NL63	KY674915	N07-6B	USA	2016
HCoV-NL63	KY674916	N07-64B	USA	2016
HCoV-NL63	LC488390	SGH-15	Japan	2017
HCoV-NL63	LC488389	SGH-18	Japan	2018
HCoV-NL63	MK334043	GD02	China	2018
HCoV-NL63	MK334044	GD03	China	2018
HCoV-NL63	MK334046	GD01	China	2018
HCoV-NL63	MN026166	KLF01	Kenya	2018
HCoV-NL63	DQ445911[§]	Amsterdan057	Netherlands	2002
HCoV-NL63	KM055646[§]	990A	China	2008
HCoV-NL63	KX179496[§]	Haiti4	Haiti	2015
HCoV-NL63	MK334047[§]	GD04	China	2018
HCoV-NL63	LC488388[§]	SGH-24	Japan	2018
HCoV-NL63	JQ765563[§]	9	USA	2009
HCoV-NL63	KF530112	0111-25	USA	2001
HCoV-NL63	DQ445912	Netherlands496	Netherlands	2003
HCoV-NL63	JQ765569	232	USA	2005
HCoV-NL63	JQ765570	235	USA	2005
HCoV-NL63	KM055643	971A	China	2008
HCoV-NL63	KX179500	UF-2	USA	2015
HCoV-NL63	KY829118	N07-262B	USA	2015
HCoV-NL63	KY554971	N07-468B176X	USA	2016
HCoV-NL63	KY554969	N07-196B	USA	2016
HCoV-NL63	MK334045	GD05	China	2018
IAV-H3N2	CY112444[*&]	6447	Switzerland	1991
IAV-H3N2	CY113773*	31	Spain	1991
IAV-H3N2	CY113653	G12	Spain	1991

IAV-H3N2	CY112452&	20	Sweden	1991
IAV-H3N2	CY114469	261	UnitedKingdom	1991
IAV-H3N2	CY113749*	938	Netherlands	1991
IAV-H3N2	CY113925	5957	Netherlands	1991
IAV-H3N2	CY113829*	467	France	1991
IAV-H3N2	CY113781	320	France	1991
IAV-H3N2	CY035222&	5	Italy	1991
IAV-H3N2	CY113661&	816	Netherlands	1991
IAV-H3N2	CY113573&	1149	France	1991
IAV-H3N2	CY113557&	260	UnitedKingdom	1991
IAV-H3N2	CY113917*	13	Sweden	1992
IAV-H3N2	CY112476&	218	Finland	1992
IAV-H3N2	CY112581*	1/3/1900	Netherlands	1992
IAV-H3N2	CY112468*	1285	Netherlands	1992
IAV-H3N2	CY112484	220	Finland	1992
IAV-H3N2	CY112508	4/7/2175	Netherlands	1992
IAV-H3N2	CY113765	3129	Netherlands	1992
IAV-H3N2	CY113909	12	Sweden	1992
IAV-H3N2	CY113877*&	9/5/1901	France	1992
IAV-H3N2	CY112460&	4112	Netherlands	1992
IAV-H3N2	CY112532	8	Sweden	1992
IAV-H3N2	CY113757	3126	Netherlands	1992
IAV-H3N2	CY112492	247	Finland	1992
IAV-H3N2	CY113677&	32	China	1992
IAV-H3N2	CY113901*&	1/7/1900	Sweden	1992
IAV-H3N2	CY113725	819	Netherlands	1992
IAV-H3N2	CY112516&	C273	Japan	1992
IAV-H3N2	CY003712&	14	HongKong	1992
IAV-H3N2	CY121325	15	China	1992
IAV-H3N2	CY114021*&	G109	Spain	1993
IAV-H3N2	CY012120&	756	USA	1993
IAV-H3N2	CY112556&	2/9/1962	France	1993
IAV-H3N2	CY113965	5458	Netherlands	1993
IAV-H3N2	CY006347	58	China	1993
IAV-H3N2	CY112653	6	Japan	1993
IAV-H3N2	CY114117	9	China	1993
IAV-H3N2	CY113957	4	Japan	1993
IAV-H3N2	CY114109&	160	UnitedKingdom	1993
IAV-H3N2	CY113989	1815	France	1993
IAV-H3N2	CY112693&	62	France	1993
IAV-H3N2	CY113997	8/13/1964	France	1993
IAV-H3N2	CY113981*&	11/2/1901	France	1993
IAV-H3N2	CY112637&	2219	Norway	1993
IAV-H3N2	CY112613&	241	Netherlands	1993

IAV-H3N2	CY113973&	25	China	1993
IAV-H3N2	CY012224*	657	USA	1994
IAV-H3N2	CY006339&	58	China	1994
IAV-H3N2	CY112701&	1/7/1900	England	1994
IAV-H3N2	CY114157&	1/18/1900	Netherlands	1994
IAV-H3N2	CY112709&	1	HongKong	1994
IAV-H3N2	CY011896	719	USA	1994
IAV-H3N2	CY010988*&	733	USA	1994
IAV-H3N2	CY002272*&	24	USA	1995
IAV-H3N2	CY017299*	701	USA	1995
IAV-H3N2	CY112781&	55	HongKong	1995
IAV-H3N2	CY112821&	359	China	1995
IAV-H3N2	CY011368	703	USA	1995
IAV-H3N2	CY112805&	1/5/1900	Sweden	1995
IAV-H3N2	CY114197	3	HongKong	1995
IAV-H3N2	CY112797*&	271	Netherlands	1995
IAV-H3N2	CY116589*	178	Netherlands	1995
IAV-H3N2	CY112789	933	China	1995
IAV-H3N2	CY114165&	338	Finland	1995
IAV-H3N2	CY114213*&	2279	France	1995
IAV-H3N2	CY038503&	3	Italy	1995
IAV-H3N2	CY114269*	434	HongKong	1996
IAV-H3N2	CY010628	608	USA	1996
IAV-H3N2	CY114237*&	20	HongKong	1996
IAV-H3N2	CY112861*&	491	France	1996
IAV-H3N2	CY114285*&	91	Netherlands	1996
IAV-H3N2	CY114245*	42	HongKong	1996
IAV-H3N2	CY114277&	11/15/1904	France	1996
IAV-H3N2	CY010716	631	USA	1996
IAV-H3N2	CY114229&	3958	Switzerland	1996
IAV-H3N2	CY011816	652	USA	1996
IAV-H3N2	CY009476*&	565	USA	1996
IAV-H3N2	CY114301*&	300	Netherlands	1997
IAV-H3N2	CY006235&	505	USA	1997
IAV-H3N2	CY036847*&	1	Italy	1997
IAV-H3N2	CY112837&	1	HongKong	1997
IAV-H3N2	CY112869&	1/21/1900	Norway	1997
IAV-H3N2	CY001477&	240	USA	1998
IAV-H3N2	CY114309*&	462	Netherlands	1998
IAV-H3N2	CY112909	10	Russia	1999
IAV-H3N2	CY090885*	NHRC0001	USA	1999
IAV-H3N2	CY090893	NHRC0001	USA	1999
IAV-H3N2	CY002112*&	6/21/1905	USA	1999
IAV-H3N2	CY121424	32	USA	1999

IAV-H3N2	CY090901	NHRC0001	USA	1999
IAV-H3N2	CY000457	149	USA	1999
IAV-H3N2	CY077848[&]	301	Netherlands	1999
IAV-H3N2	CY121408*[&]	1	Russia	2000
IAV-H3N2	CY000689	173	USA	2000
IAV-H3N2	CY114317*[&]	3	Netherlands	2000
IAV-H3N2	CY090909[&]	NHRC0001	USA	2000
IAV-H3N2	CY112925	118	Netherlands	2001
IAV-H3N2	CY000185*[&]	83	USA	2001
IAV-H3N2	CY114325*[&]	124	Netherlands	2001
IAV-H3N2	CY091093*	NHRC0001	USA	2002
IAV-H3N2	CY037343[&]	1	Italy	2002
IAV-H3N2	CY003176[&]	414	USA	2002
IAV-H3N2	CY114341[&]	120	Netherlands	2002
IAV-H3N2	CY088483[&]	411	China	2002
IAV-H3N2	CY091101	NHRC0001	USA	2002
IAV-H3N2	CY091109	NHRC0001	USA	2002
IAV-H3N2	CY121448*[&]	445	China	2003
IAV-H3N2	CY100594[&]	InDRE2662	Mexico	2003
IAV-H3N2	CY088438[&]	8/6/1901	UnitedKingdom	2003
IAV-H3N2	CY000873	194	USA	2003
IAV-H3N2	CY112965*	88	Netherlands	2003
IAV-H3N2	CY032961*	844	Mexico	2003
IAV-H3N2	CY092201*	NHRC0001	USA	2003
IAV-H3N2	CY090933	NHRC0001	USA	2003
IAV-H3N2	CY000001[&]	61A	USA	2003
IAV-H3N2	CY088006	50	UnitedKingdom	2003
IAV-H3N2	CY112949*[&]	20	Netherlands	2003
IAV-H3N2	CY112973*	109	Netherlands	2003
IAV-H3N2	CY091213*	NHRC0004	USA	2003
IAV-H3N2	CY100618	InDRE835	Mexico	2003
IAV-H3N2	CY090949	NHRC0002	USA	2003
IAV-H3N2	CY002104	1/31/1900	USA	2003
IAV-H3N2	CY092209	NHRC0001	USA	2003
IAV-H3N2	CY037351*[&]	14	Italy	2003
IAV-H3N2	CY091461	NHRC0006	USA	2003
IAV-H3N2	CY090957	NHRC0001	USA	2003
IAV-H3N2	CY034108*	3	USA	2003
IAV-H3N2	CY090965	NHRC0001	USA	2003
IAV-H3N2	CY090941	NHRC0001	USA	2003
IAV-H3N2	CY114349[&]	170	Finland	2003
IAV-H3N2	CY112989[&]	132	Netherlands	2004
IAV-H3N2	CY040082[&]	9	Taiwan	2004
IAV-H3N2	CY038567[&]	HKU2	HongKong	2004

IAV-H3N2	CY114373	1/7/1900	USA	2004
IAV-H3N2	CY039047*	HKU40	HongKong	2004
IAV-H3N2	CY002288	207	USA	2004
IAV-H3N2	CY090973	NHRC0001	USA	2004
IAV-H3N2	CY092108&	NHRC0001	USA	2004
IAV-H3N2	CY038575*	HKU8	HongKong	2004
IAV-H3N2	CY091117	NHRC0002	USA	2004
IAV-H3N2	CY090997*&	NHRC0002	USA	2005
IAV-H3N2	CY091125*	NHRC0001	USA	2005
IAV-H3N2	CY091133*	NHRC0001	USA	2005
IAV-H3N2	CY100554	InDRE2118	Mexico	2005
IAV-H3N2	CY091525	NHRC0006	USA	2005
IAV-H3N2	CY112997	7/1/1901	Netherlands	2005
IAV-H3N2	CY090981*	NHRC0001	USA	2005
IAV-H3N2	CY100562	InDRE2160	Mexico	2005
IAV-H3N2	CY091509	NHRC0004	USA	2005
IAV-H3N2	CY172199	1002	USA	2005
IAV-H3N2	CY091533	NHRC0007	USA	2005
IAV-H3N2	CY091021	NHRC0002	USA	2005
IAV-H3N2	CY039175&	HKU44	HongKong	2005
IAV-H3N2	CY114381*&	67	USA	2005
IAV-H3N2	CY172343*&	1023	USA	2006
IAV-H3N2	CY114397	12/28/1900	Netherlands	2006
IAV-H3N2	CY100602&	InDRE29	Mexico	2006
IAV-H3N2	CY114389*&	42	Netherlands	2006
IAV-H3N2	CY012792	4	USA	2006
IAV-H3N2	CY025843	UR06-0510	USA	2007
IAV-H3N2	CY026147*	UR06-0023	USA	2007
IAV-H3N2	CY025603*	UR06-0471	USA	2007
IAV-H3N2	CY027571	UR06-0534	USA	2007
IAV-H3N2	CY025739*	UR06-0118	USA	2007
IAV-H3N2	CY172831*	1090	USA	2007
IAV-H3N2	CY030197	UR06-0600	USA	2007
IAV-H3N2	CY091829&	15	China	2007
IAV-H3N2	CY026771	UR06-0252	USA	2007
IAV-H3N2	CY114405*&	69	Netherlands	2007
IAV-H3N2	CY040130	1452	Taiwan	2007
IAV-H3N2	CY040122	5/31/2097	Taiwan	2007
IAV-H3N2	CY025755	UR06-0150	USA	2007
IAV-H3N2	CY025907	UR06-0370	USA	2007
IAV-H3N2	CY025859	UR06-0545	USA	2007
IAV-H3N2	CY025747&	UR06-0480	USA	2007
IAV-H3N2	CY027795	UR06-0200	USA	2007
IAV-H3N2	CY025931	UR06-0453	USA	2007

IAV-H3N2	CY030205*	UR06-0605	USA	2007
IAV-H3N2	CY121528	1/3/1900	USA	2007
IAV-H3N2	CY114413	348	Netherlands	2007
IAV-H3N2	CY069357&	WRAIR1059P	Japan	2008
IAV-H3N2	CY091835*&	314	China	2008
IAV-H3N2	CY173175&	1141	USA	2008
IAV-H3N2	CY147627&	24009	Mexico	2008
IAV-H3N2	CY036999*	UR07-0140	USA	2008
IAV-H3N2	CY091855*	578	China	2008
IAV-H3N2	CY037303*&	AF02	Qatar	2008
IAV-H3N2	CY040098&	70120	Taiwan	2008
IAV-H3N2	CY035190	PIT43	USA	2008
IAV-H3N2	CY037311*&	AF03	Kuwait	2008
IAV-H3N2	CY173271	1154	USA	2008
IAV-H3N2	CY173183	1142	USA	2008
IAV-H3N2	CY093319&	WRAIR1256P	Kyrgyzstan	2008
IAV-H3N2	CY037847	UR07-0136	USA	2008
IAV-H3N2	CY037319&	AF05	Korea	2008
IAV-H3N2	CY037511	UR07-0025	USA	2008
IAV-H3N2	CY037567	UR07-0081	USA	2008
IAV-H3N2	CY038839	UR07-0076	USA	2008
IAV-H3N2	CY044461	5	USA	2008
IAV-H3N2	CY038495	PIT39	USA	2008
IAV-H3N2	CY068694*	VRDL183	USA	2009
IAV-H3N2	CY069517*	WRAIR1240	USA	2009
IAV-H3N2	CY173559	1194	USA	2009
IAV-H3N2	CY062348&	N05371	Jordan	2009
IAV-H3N2	CY064887	VRDL158	USA	2009
IAV-H3N2	CY093295	WRAIR1251P	USA	2009
IAV-H3N2	CY093343	WRAIR1558P	USA	2009
IAV-H3N2	CY050090*&	2157	China	2009
IAV-H3N2	CY053672*&	707	Russia	2009
IAV-H3N2	CY121776&	97	Finland	2009
IAV-H3N2	CY062349&	N13417	Egypt	2009
IAV-H3N2	CY093303*&	WRAIR1252P	Iraq	2009
IAV-H3N2	CY147723&	10/10/1965	Mexico	2009
IAV-H3N2	CY093240*	WRAIR1171P	SouthKorea	2009
IAV-H3N2	CY053660	1/31/1900	Russia	2009
IAV-H3N2	CY093263	WRAIR1242P	USA	2009
IAV-H3N2	CY089749	75	USA	2009
IAV-H3N2	CY069461&	WRAIR1145P	USA	2009
IAV-H3N2	CY106680*&	H090-707-V10	HongKong	2009
IAV-H3N2	CY113021&	3/9/1900	Netherlands	2009
IAV-H3N2	CY062334	N04877	Egypt	2009

IAV-H3N2	CY093351[&]	WRAIR1561P	Kuwait	2009
IAV-H3N2	CY093327	WRAIR1258P	USA	2009
IAV-H3N2	CY069317*[&]	WRAIR1037P	Japan	2009
IAV-H3N2	CY062346	N12479	Egypt	2009
IAV-H3N2	CY093359	WRAIR1562P	USA	2009
IAV-H3N2	CY069501	WRAIR1177P	USA	2009
IAV-H3N2	CY068177	VRDL166	USA	2009
IAV-H3N2	CY091839	2/26/1901	China	2009
IAV-H3N2	CY114509*	34	Netherlands	2010
IAV-H3N2	CY091845*[&]	460	China	2010
IAV-H3N2	CY093383	WRAIR1753P	USA	2010
IAV-H3N2	CY121213	119	USA	2010
IAV-H3N2	CY167282[&]	F2008c07	USA	2010
IAV-H3N2	CY093503[&]	WRAIR3571N	Mexico	2010
IAV-H3N2	CY093559	WRAIR4139N	Mexico	2010
IAV-H3N2	CY093391*[&]	WRAIR2379N	Serbia	2010
IAV-H3N2	CY114501[&]	9	Netherlands	2010
IAV-H3N2	CY093224*	WRAIR0300	USA	2010
IAV-H3N2	CY111009[&]	7	Canada	2010
IAV-H3N2	CY070967	20342	USA	2010
IAV-H3N2	CY121800*[&]	1	USA	2010
IAV-H3N2	CY084299	AF2692	USA	2010
IAV-H3N2	CY111007*	3	Canada	2010
IAV-H3N2	CY099953[&]	94	China	2011
IAV-H3N2	CY114421[&]	063	Netherlands	2011
IAV-H3N2	CY091557*	NHRC0001	USA	2011
IAV-H3N2	CY111126	DOA01	USA	2011
IAV-H3N2	CY091565*	NHRC0001	USA	2011
IAV-H3N2	CY091573	NHRC0002	USA	2011
IAV-H3N2	CY091581	NHRC0001	USA	2011
IAV-H3N2	CY093567[&]	WRAIR4307N	Russia	2011
IAV-H3N2	CY111000*[&]	21	Canada	2011
IAV-H3N2	CY114553	RII65	Russia	2011
IAV-H3N2	CY134656	3100	USA	2012
IAV-H3N2	CY141268	11/26/1908	USA	2012
IAV-H3N2	CY130200[&]	3060	Japan	2012
IAV-H3N2	CY134663	3107	USA	2012
IAV-H3N2	CY264633*	9	USA	2012
IAV-H3N2	CY134659	3103	USA	2012
IAV-H3N2	CY134640	3084	USA	2012
IAV-H3N2	CY114548*[&]	RII03	Russia	2012
IAV-H3N2	CY120885	3000	USA	2012
IAV-H3N2	CY114533*	RII01	Russia	2012
IAV-H3N2	CY134638	6/8/1908	USA	2012

IAV-H3N2	CY130194	3058	USA	2012
IAV-H3N2	CY120890	3005	USA	2012
IAV-H3N2	CY141209	3194	USA	2012
IAV-H3N2	CY134660	6/30/1908	USA	2012
IAV-H3N2	CY120889	3004	Japan	2012
IAV-H3N2	CY134636	3080	USA	2012
IAV-H3N2	CY171063[&]	YGA04006	USA	2012
IAV-H3N2	CY141181	3166	USA	2012
IAV-H3N2	CY134748*	JMM3-1	USA	2012
IAV-H3N2	CY116636*[&]	GNCDC0485	Georgia	2012
IAV-H3N2	CY134657	6/27/1908	USA	2012
IAV-H3N2	CY134649	3093	USA	2012
IAV-H3N2	CY125691[&]	947	China	2012
IAV-H3N2	CY114538*	RII08	Russia	2012
IAV-H3N2	CY125791*	DOA94	USA	2012
IAV-H3N2	CY110774	59	Russia	2012
IAV-H3N2	CY120872[&]	3/5/1908	USA	2012
IAV-H3N2	CY120883	3/16/1908	USA	2012
IAV-H3N2	CY141282	3267	USA	2013
IAV-H3N2	CY163423	5/27/1909	USA	2013
IAV-H3N2	CY141182	3167	USA	2013
IAV-H3N2	CY163424	3436	USA	2013
IAV-H3N2	CY141184	9/3/1908	USA	2013
IAV-H3N2	CY186243*	JMM179	USA	2013
IAV-H3N2	CY163425	5/29/1909	USA	2013
IAV-H3N2	CY147294	4/24/1909	Korea	2013
IAV-H3N2	CY141213	10/2/1908	USA	2013
IAV-H3N2	CY187381	3713	Japan	2013
IAV-H3N2	CY147295[&]	3403	Japan	2013
IAV-H3N2	CY141191	3176	USA	2013
IAV-H3N2	CY141216	3201	USA	2013
IAV-H3N2	CY163426	3438	USA	2013
IAV-H3N2	CY163428	3440	USA	2013
IAV-H3N2	CY141219	3204	USA	2013
IAV-H3N2	CY163429	3441	USA	2013
IAV-H3N2	CY141205[&]	3190	USA	2013
IAV-H3N2	CY141202	3187	USA	2013
IAV-H3N2	CY193271	4018	USA	2014
IAV-H3N2	CY189822	3902	USA	2014
IAV-H3N2	CY193397	4144	USA	2014
IAV-H3N2	CY193346	4093	USA	2014
IAV-H3N2	CY187645	3/15/1910	USA	2014
IAV-H3N2	CY193270	4017	SouthKorea	2014
IAV-H3N2	CY187675[&]	3757	Japan	2014

IAV-H3N2	CY193400	4147	USA	2014
IAV-H3N2	CY193474	7/22/1911	USA	2014
IAV-H3N2	CY187383[&]	3715	USA	2014
IAV-H3N2	CY193471	4218	USA	2014
IAV-H3N2	CY187709	3791	USA	2014
IAV-H3N2	CY187712	3794	USA	2014
IAV-H3N2	CY193348	4095	USA	2014
IAV-H3N2	CY187382	3/2/1910	USA	2014
IAV-H3N2	CY193259	4006	USA	2014
IAV-H3N2	CY187698[&]	3780	Korea	2014
IAV-H3N2	CY193405	4152	USA	2014
IAV-H3N2	CY193266	4013	USA	2014
IAV-H3N2	CY193378	4125	USA	2014
IAV-H3N2	CY193361	4108	USA	2014
IAV-H3N2	CY193406	4153	USA	2014
IAV-H3N2	CY187647	3729	USA	2014
IAV-H3N2	CY193413	4160	USA	2014
IAV-H3N2	CY193262	12/22/1910	USA	2014
IAV-H3N2	CY193263	4010	USA	2014
IAV-H3N2	CY193320	4067	USA	2014
IAV-H3N2	CY193349	4096	USA	2014
IAV-H3N2	CY193395	4142	USA	2014
IAV-H3N2	CY193358	4105	USA	2014
IAV-H3N2	CY193864	4611	USA	2015
IAV-H3N2	CY193825	4572	USA	2015
IAV-H3N2	CY193827	7/9/1912	USA	2015
IAV-H3N2	CY193916	4663	USA	2015
IAV-H3N2	CY193863	4610	USA	2015
IAV-H3N2	CY193890	4637	USA	2015
IAV-H3N2	CY193826[&]	7/8/1912	USA	2015
IAV-H3N2	CY208122	4889	USA	2015
IAV-H3N2	CY193862	4609	USA	2015
IAV-H3N2	CY208126	4893	USA	2015
IAV-H3N2	CY193917	4664	USA	2015
IAV-H3N2	CY193818	4565	USA	2015
IAV-H3N2	CY208123	4890	Japan	2015
IAV-H3N2	CY193891	4638	USA	2015
IAV-H3N2	CY210030	5459	USA	2016
IAV-H3N2	CY210815*	33	USA	2016
IAV-H3N2	CY209873	10/1/1914	USA	2016
IAV-H3N2	CY208768*	68	USA	2016
IAV-H3N2	CY210306	2/6/1900	USA	2016
IAV-H3N2	CY210393	8	USA	2016
IAV-H3N2	CY208784	2/15/1900	USA	2016

IAV-H3N2	CY210687*	16	USA	2016
IAV-H3N2	CY210353*	93	USA	2016
IAV-H3N2	CY218982	49	USA	2016
IAV-H3N2	CY210171	24	USA	2016
IAV-H3N2	CY210049	5478	USA	2016
IAV-H3N2	CY217906*	116	USA	2016
IAV-H3N2	CY208201	4968	USA	2016
IAV-H3N2	CY210703*	97	USA	2016
IAV-H3N2	CY208872	25	USA	2016
IAV-H3N2	CY210298*	103	USA	2016
IAV-H3N2	CY216105	4/27/1900	USA	2016
IAV-H3N2	CY210322	39	USA	2016
IAV-H3N2	CY208808	23	USA	2016
IAV-H3N2	CY210671	92	USA	2016
IAV-H3N2	CY210329	30	USA	2016
IAV-H3N2	CY211708	26	USA	2016
IAV-H3N2	CY210053	5482	SouthKorea	2016
IAV-H3N2	CY208744	24	USA	2016
IAV-H3N2	CY211650	18	USA	2016
IAV-H3N2	CY211716*	17	USA	2016
IAV-H3N2	CY210401	104	USA	2016
IAV-H3N2	CY208736	20	USA	2016
IAV-H3N2	CY208816	183	USA	2016
IAV-H3N2	CY208212	8/18/1913	USA	2016
IAV-H3N2	CY210807	63	USA	2016
IAV-H3N2	CY210416	33	USA	2016
IAV-H3N2	CY211697*	31	USA	2016
IAV-H3N2	CY211474	26	USA	2016
IAV-H3N2	CY208776[&]	46	USA	2016
IAV-H3N2	CY210018	11/29/1914	USA	2016
IAV-H3N2	CY210631*	19	USA	2016
IAV-H3N2	CY210615	40	USA	2016
IAV-H3N2	CY217914	87	USA	2016
IAV-H3N2	CY210727	24	USA	2016
IAV-H3N2	CY218222*	3	USA	2017
IAV-H3N2	CY261583*	42	USA	2017
IAV-H3N2	CY216033*	4	USA	2017
IAV-H3N2	CY216177*	2	USA	2017
IAV-H3N2	CY264409*	2/28/1900	USA	2017
IAV-H3N2	CY261479*	30	USA	2017
IAV-H3N2	CY216137*	1/7/1900	USA	2017
IAV-H3N2	CY216185	3	USA	2017
IAV-H3N2	CY216425*	8	USA	2017
IAV-H3N2	CY216353*	7/9/1905	USA	2017

IAV-H3N2	CY264441*	44	USA	2017
IAV-H3N2	CY261495	15	USA	2017
IAV-H3N2	CY215889	8	USA	2017
IAV-H3N2	CY261280	267	USA	2017
IAV-H3N2	CY216377*	4	USA	2017
IAV-H3N2	CY216657	7	USA	2017
IAV-H3N2	CY216569	2	USA	2017
IAV-H3N2	CY261543	26	USA	2017
IAV-H3N2	CY264497	40	USA	2017
IAV-H3N2	CY261264*	43	USA	2017
IAV-H3N2	CY216193	3	USA	2017
IAV-H3N2	CY216473*	3	USA	2017
IAV-H3N2	CY264361	97	USA	2017
IAV-H3N2	CY216345	6	USA	2017
IAV-H3N2	CY216441	15	USA	2017
IAV-H3N2	CY261431*	33	USA	2017
IAV-H3N2	CY264457	42	USA	2017
IAV-H3N2	CY264513	31	USA	2017
IAV-H3N2	CY261551*	36	USA	2017
IAV-H3N2	CY264353	340	USA	2017
IAV-H3N2	CY216145	4	USA	2017
IAV-H3N2	CY216385	3	USA	2017
IAV-H3N2	CY261343	38	USA	2017
IAV-H3N2	CY216273	3	USA	2017
IAV-H3N2	CY261511	32	USA	2017
IAV-H3N2	CY216409	2	USA	2017
IAV-H3N2	CY263144	28	USA	2017
IAV-H3N2	CY216785*	4	USA	2017
IAV-H3N2	CY261623*	31	USA	2017
IAV-H3N2	CY216801*	5	USA	2017
IAV-H3N2	CY215881[&]	1/2/1900	USA	2017
IAV-H3N2	CY264521	42	USA	2017
IAV-H3N2	CY265149	44	USA	2017
IAV-H3N2	CY216737	1/23/1900	USA	2017
IAV-H3N2	CY261415	95	USA	2017
IAV-H3N2	CY216729	6	USA	2017
IAV-H3N2	CY218929*	3	USA	2017
IAV-H3N2	CY216041*	2	USA	2017
IAV-H3N2	CY265213	33	USA	2017
IAV-H3N2	CY261320	26	USA	2017
IAV-H3N2	CY261607	43	USA	2017
IAV-H3N2	CY216577	19	USA	2017
IAV-H3N2	CY216689	5	USA	2017
IAV-H3N2	CY216641	4	USA	2017

IAV-H3N2	CY216489	1	USA	2017
IAV-H3N2	CY261439	34	USA	2017
IAV-H3N2	CY261232	48	USA	2017
IAV-H3N2	CY261359	126	USA	2017
IAV-H3N2	MK382012[&]	7024	Germany	2018
IAV-H3N2	MK382032[&]	7044	Spain	2018
IAV-H3N2	MH637438[&]	P0287	USA	2018
IAV-H3N2	MH701580	6/9/2088	USA	2018
IAV-H3N2	MH885273[&]	6859	SouthKorea	2018
IAV-H3N2	MK400723[&]	7127	UnitedKingdom	2018
IAV-H3N2	MN826673	1287	SouthKorea	2018
IAV-H3N2	MN538407[&]	71	Canada	2018
IAV-H3N2	MH700956	BRD41925N	USA	2018
IAV-H3N2	MH778984	3/2/1918	USA	2018
IAV-H3N2	MK898400	NHRC37141	USA	2018
IAV-H3N2	MH778968	6620	USA	2018
IAV-H3N2	MH701636[&]	FDX70344	Japan	2018
IAV-H3N2	MN538395	98	Canada	2018
IAV-H3N2	MN826665	1320	SouthKorea	2018
IAV-H3N2	MH885217	6803	USA	2018
IAV-H3N2	MH294904	6538	USA	2018
IAV-H3N2	MN594902[&]	5/31/1905	China	2018
IAV-H3N2	MH701028[&]	RVT0142	Bahrain	2018
IAV-H3N2	MH778966	6618	USA	2018
IAV-H3N2	MH797814	PV00377	USA	2018
IAV-H3N2	MN538761	85	Canada	2018
IAV-H3N2	MN826641	1343	SouthKorea	2018
IAV-H3N2	MH779056	6708	USA	2018
IAV-H3N2	MN538372	30	Canada	2018
IAV-H3N2	MH294939	6573	USA	2018
IAV-H3N2	MN170088	9138	USA	2019
IAV-H3N2	MN169448	9038	USA	2019
IAV-H3N2	MN170120	9142	USA	2019
IAV-H3N2	MN154249	9741	USA	2019
IAV-H3N2	MN170160[&]	9147	USA	2019
IAV-H3N2	MK869195[&]	8602	Italy	2019

* Subset of IAV-H3N2-HA sequences in selection pressure analyses
[†] Laboratory strains
[§] Sequences taken out due to detection of recombination event with >3 methods p<0.5
[#] Sequences taken out from pressure analyses
[&] Sequences used in subsampled dataset
