# Supplementary material for: The evolutionary dynamics of endemic human coronaviruses
Source: Virus Evol. 2021 Mar 20;7(1):veab020. doi: 10.1093/ve/veab020 (PMC7980080; doi:10.1093/ve/veab020)
Supplement: veab020_Supplementary_Data [file veab020_supplementary_data.zip › FigureS1.pdf]

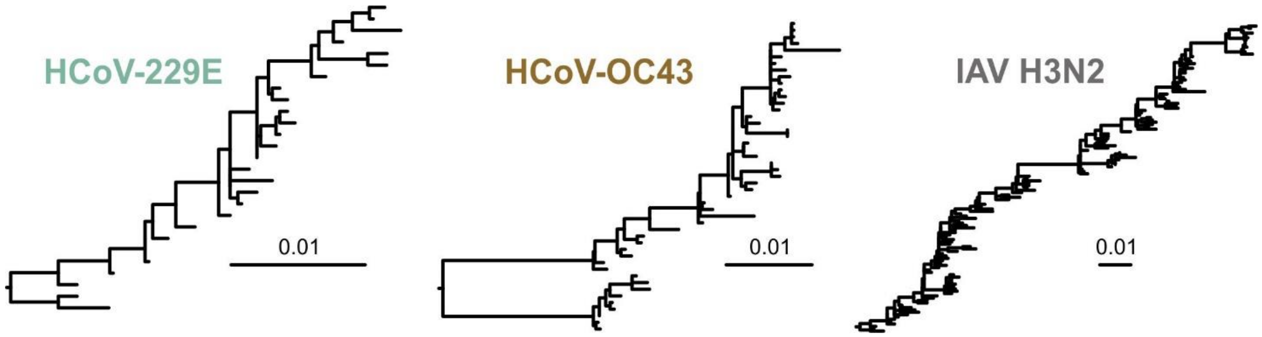

Figure S1. Maximum likelihood phylogenies of HCoV-229E S, HCoV-OC43 S, and IAV H3N2 HA subsampled datasets. Scale bars indicate number of nucleotide substitutions per site. Sequences used are detailed in Table S1.
